# Social support and online interaction and their links to psychosocial well‐being among Nordic adolescents: Integrating variable‐centered and person‐centered approaches

**DOI:** 10.1111/jora.13058

**Published:** 2025-01-24

**Authors:** Jasmine Gustafsson, Inga Jasinskaja‐Lahti, Hanna Konttinen, Nina Simonsen, Petra Löfstedt, Nelli Lyyra

**Affiliations:** ^1^ Social Psychology, Faculty of Social Sciences University of Helsinki Helsinki Finland; ^2^ Public Health Research Program Folkhälsan Research Center Helsinki Finland; ^3^ Department of Public Health University of Helsinki Helsinki Finland; ^4^ School of Public Health and Community Medicine, Institute of Medicine Gothenburg University Göteborg Sweden; ^5^ Faculty of Sport and Health Sciences University of Jyväskylä Jyväskylä Finland

**Keywords:** latent profile analysis, Nordic countries, psychosomatic health, relationship quality, social media, well‐being

## Abstract

The Nordic countries are among the most digitally advanced societies in the world. Past research suggests that both social support offline and interaction online are linked to adolescent psychological adjustment. However, less is known regarding the complex implications of distinctive sources of social support offline and online interaction for a broader range of indices of adolescent psychosocial well‐being, including its contemporary forms such as social media addiction. This study utilized two methodological approaches to examine the social components and determinants of psychosocial well‐being (psychosomatic complaints and problematic social media use) among 22,384 Nordic adolescents aged between 11 and 15 years. A variable‐centered approach focused on examining perceived support from four sources (family, peers, teachers, and classmates), preference for online interaction, and intensity of online interaction as predictors of psychosocial well‐being. Concurrently, a person‐centered approach was utilized to explore the subgroups at risk of psychosocial ill‐being. In the variable‐centered analysis, lower support from family, teachers, and classmates, higher preference for online interaction, and higher intensity of online interaction with “online contacts” (i.e., interaction partners met online) were linked to higher levels of psychosomatic complaints and more problematic social media use. Additionally, lower peer support was associated with increased psychosomatic complaints, while greater intensity of online interaction with “offline contacts” (e.g., close friends, parents) was related to more problematic social media use. The person‐centered analysis identified five profiles of adolescents: (1) “Multiply supported online users” (56%), (2) “Primarily (family‐peer) supported high online users” (22%), (3) “Non‐supported online users” (13%), (4) “Primarily non‐supported online users” (5%), and (5) “Non‐supported high online users” (4%). Adolescents reporting higher support from multiple sources and moderate online interaction with offline contacts experienced the lowest levels of psychosomatic complaints and the least problematic social media use, while the other profiles exhibited more complex and less optimal psychosocial well‐being. In conclusion, these findings suggest that adolescents benefit most from robust social support offline across multiple social networks, but strong support from certain sources (teachers, classmates) can somewhat mitigate the adverse effects of low support from other sources (family, peers). The relationship between online interaction and psychosocial well‐being is contingent on the level of social support.

## INTRODUCTION

Adolescence is widely recognized as a crucial stage of life, given that mental health problems often surface during this period (Solmi et al., 2022) and can impact long‐term well‐being well into adulthood (Copeland et al., 2015; Schlack et al., 2021). In Europe, there is a significant concern regarding mental health problems among youth, with 16% of those aged 10–19 years experiencing conditions such as depression and anxiety, surpassing the global average of 13% for the same age group (UNICEF, 2021). In recent years, there has also been an increase in the prevalence of reoccurring multiple psychosomatic health complaints, such as feeling low and experiencing headaches, among adolescents aged 11, 13, and 15 years across 44 countries, with the prevalence rising from about one third (33%) in 2014 to almost half (44%) in 2022 (Cosma et al., 2023). Although the Nordic countries are frequently lauded for their promotion of health through public health policies (Raphael, 2014), the paradoxical rise in mental health problems among adolescents in these nations particularly (Potrebny et al., 2017) calls for closer examination of the factors contributing to and protective resources for health challenges.

Psychosocial well‐being is a multifaceted construct that encompasses an individual's mental, emotional, and social health (Eiroa‐Orosa, 2020; Larson, 1996). It reflects not only positive functioning and the absence of disturbances but also signifies the lack of maladaptive behavior patterns (Sinha, 2011). Over the last decade, the rapid expansion of the Internet and social networking sites has sparked concerns about their potential implications for psychosocial well‐being, specifically among children and adolescents. Research indicates that high digital engagement can contribute to issues such as depression and anxiety, particularly among youth (Cunningham et al., 2021; Meier & Reinecke, 2021). The unique social context of the Internet has also given rise to a new form of psychobehavioral pathology—problematic social media use—which is characterized by a persistent fixation on social media, an inability to disengage, and continued use despite its adverse effects on daily habits (e.g., lack of sleep), cognitive processes (e.g., attention deficits), and social functioning (e.g., relationships with family, friends, and school) (Van den Eijnden et al., 2016). As users navigate virtual spaces, the immediacy of online availability, along with the intrinsically rewarding nature of online interactions—such as receiving “likes” or comments—can lead to an overreliance on digital validation and social feedback (Nesi & Prinstein, 2015). According to a cross‐national survey by Boer et al. (2020), which utilized data from the 2017/18 cycle of the Health Behaviour in School‐aged Children (HBSC) study, Nordic adolescents exhibit rates of problematic social media use ranging from 4% in Denmark to 10% in Finland. This contrasts with an average rate of 7% observed across 29 countries.

Adolescents' psychosocial well‐being and overall development are significantly impacted by their social connections (Orben et al., 2020). Traditionally, studies on social support in adolescence have restricted their focus to offline interactions. Research has shown that adolescents are expected to benefit from offline social support from various sources such as family, peers, or teachers, although the relative importance and interactions of these sources may also vary (Chu et al., 2010; Rueger et al., 2016), yielding different health outcomes (Chan et al., 2022). As youth increasingly engage with digital technologies, online interaction has emerged not only as a potential source of ill‐being, but also as a novel channel for social support, allowing individuals to form new bonds across time and space. Virtual connectivity not only allows the maintenance of existing relationships—where evidence suggests that stronger social networks offline correlate with higher online engagement (Domahidi, 2018)—but also facilitates the forming of new bonds, especially among those who feel dissatisfied with the support they receive from their current offline contacts (Chung, 2013; Leung, 2011). However, interaction that takes place online may differ from adolescents' offline social worlds (Nesi et al., 2018). Recent evidence emphasizes the need to consider individual differences in how online interaction impacts well‐being, suggesting that social media use may be beneficial for some groups, detrimental for others, and neutral for certain individuals (Beyens et al., 2020). This complexity emphasizes the need for a nuanced approach when considering the implications of support networks and online interaction for different individuals.

This study aimed to explore the role of social support from different sources offline in predicting two dimensions of adolescents' psychosocial well‐being (i.e., psychosomatic complaints and problematic social media use), while accounting for their online interaction behaviors. The study contributes to the field by (1) looking at multiple psychosocial outcomes, including psychosomatic complaints and problematic social media use, (2) examining these outcomes in relation to both offline social support and online interactions, and (3) employing two different methodological approaches (i.e., variable‐ and person‐centered).

### Transformative impacts of online interaction on adolescent social support

Social support encompasses the beneficial functions or resources provided by significant others to help recipients manage stress or fulfill specific needs (Cohen, 2004; Thoits, 1995). Even in the absence of stressors or adverse conditions, offline social support has been demonstrated to contribute to improved well‐being by lowering the risk of depression (Rueger et al., 2016). Online interaction includes a broad range of activities that encompass not only the transmission of messages but also engagement and participation in digital spaces through various media forms, such as text, emojis, and multimedia content (Walther, 1996). Research has identified several key similarities between adolescents' online and offline interactions, highlighting how they express affection through humor and laughter, cultivate intimacy by sharing secrets and personal photos, and celebrate important milestones like birthdays, which may reinforce feelings of validation and significance (Yau & Reich, 2018). The gratification from receiving “likes” on shared content, such as photos or updates, can be rewarding (Rosenthal‐von der Pütten et al., 2019), offering a sense of validation and perceived support (Wohn et al., 2016).

However, some scholars argue that social media represents a distinct social context that differs from adolescents' offline social worlds. For instance, Nesi et al. (2018) propose a transformation framework suggesting that platforms like Instagram and Facebook may alter the nature of interactions, including social support, by providing access to broader networks, facilitating more frequent and persistent interactions, and offering features such as carefully curated self‐presentation and long‐term visibility of support requests. However, the lack of nonverbal cues and potential delays between messages—known as asynchrony—can diminish the richness of the interaction (Peterson et al., 2018; Scott et al., 2022). Moreover, social media's emphasis on quantitative social metrics, such as the number of likes, friends, or followers, and a focus on visual content may amplify users' sensitivity to feedback (Chua & Chang, 2016; de Vries et al., 2016; Schreurs et al., 2024). Nevertheless, some individuals, particularly those experiencing social anxiety, may find that these features enhance their confidence in interacting with others online (Angelini & Gini, 2023; Prizant‐Passal et al., 2016).

More recent empirical research indicates that social media predominantly plays a beneficial role in enhancing offline friendships during preadolescence and adolescence (Angelini et al., 2022, 2024; Meeus et al., 2022). For example, Angelini et al. (2022) found that adolescents' perceptions of social media features were associated with various dimensions of friendship quality. Specifically, adolescents who viewed social media as a platform for frequent connections with a wide peer network reported higher satisfaction with the instrumental support they received from friends—defined in the study as being helpful and protective. These adolescents also experienced a greater sense of companionship. Additionally, those who perceived social media as a platform for asynchronous interactions (i.e., allowing one to take time before replying) felt more capable of resolving potential conflicts with friends and experienced increased validation within their relationships. Conversely, adolescents who perceived fewer audiovisual cues reported lower levels of online social support, which was linked to a diminished sense of intimacy in their peer relationships. These findings suggest that adolescents consider social media features as vital tools for enhancing the quality of their friendships while also highlighting the complexities inherent in online interactions.

### A variable‐centered approach to social support and online interaction and their links to psychosocial well‐being

Most previous research on social support, online interaction, and psychosocial well‐being has utilized a variable‐centered approach, which is fruitful for examining associations between variables across entire samples (Howard & Hoffman, 2018; Laursen & Hoff, 2006). Extensive variable‐centered research (Bi et al., 2021; Chu et al., 2010; Rueger et al., 2016) has demonstrated positive links between perceived social support from parents, peers, and teachers and various indicators of well‐being, such as higher life satisfaction and self‐esteem, as well as lower levels of depression and anxiety. In addition, although less frequently examined, higher social support has been linked to lower problematic social media use (Boer et al., 2020; Boniel‐Nissim et al., 2022; Borraccino et al., 2022; Wong et al., 2022). Nonetheless, the importance of specific sources of support appears to vary. For instance, meta‐analyses by Chu et al. (2010) and Kim et al. (2018) found that teacher support was more strongly linked with adolescents' well‐being than support from parents or peers. In contrast, two other meta‐analyses (Heerde & Hemphill, 2018; Rueger et al., 2016) observed that support from family and peers was more influential than other support networks. These inconsistencies in the unique effects of specific support sources highlight the complex nature of social support in relation to adolescents' well‐being.

Further, studies adopting a variable‐centered approach have investigated the differences between support received or perceived through online platforms and face‐to‐face interactions. Research encompassing adolescents has demonstrated that higher levels of social support in offline settings are associated with enhanced well‐being, including higher self‐esteem (Lin et al., 2018), fewer depressive symptoms (Chen et al., 2024; Lin et al., 2018), and lower rates of problematic social media use (Angelini et al., 2022). However, these benefits do not extend to online social support in these studies. Conversely, some research that exclusively examined adolescents' online social support—excluding offline support—identified positive associations with well‐being. For example, a study by Nguyen and Ho (2022) found a very weak correlation between higher levels of online social support and increased well‐being among adolescents, particularly in terms of happiness. Similarly, Politte‐Corn et al. (2023) demonstrated that higher online social support is associated with lower levels of depressive symptoms among adolescents (aged <17 years), although this relationship did not extend to young adults. In addition, a meta‐analysis by Zhou and Cheng (2022) found that online social support was moderately linked to higher self‐esteem but was not related to depression. In contrast, Knowles (2024) reported no significant links between online social support and the prevalence of depression or anxiety among adolescents and young adults. This suggests that while offline social support appears to contribute positively to adolescent well‐being, the evidence for online social support is more limited and inconsistent.

In the online interaction literature, reviews and meta‐analyses have revealed mixed findings. These works indicate small positive or negative associations, or nonsignificant associations between various measures of online interaction and adolescents' well‐being outcomes such as depression, self‐esteem, anxiety, and distress (Best et al., 2014; Cunningham et al., 2021; Meier & Reinecke, 2021; Orben, 2020; Valkenburg et al., 2022). Higher online interaction has also been linked to higher levels of problematic social media use (Boer et al., 2020). On the other hand, other meta‐analyses (Domahidi, 2018; Frost & Rickwood, 2017) and recent empirical studies (Boer et al., 2020; Boniel‐Nissim et al., 2022) have observed that social media use, including online interaction, is associated with improved social well‐being, providing users with greater social support and enhanced social resources. Notably, the study by Boer et al. (2020) found that higher engagement in online interaction was linked to higher support from peers, while the relationship between online interaction and family support varied across different countries, showing positive associations in some contexts and negative ones in others. The nature of adolescents' online connections may also influence their well‐being. Recent evidence (Lyyra et al., 2022) suggests that frequent online interaction with offline friends is linked to better well‐being, including enhanced self‐rated health, higher life satisfaction, and lower loneliness. Conversely, online interaction with online friends and unknown people is associated with poorer well‐being outcomes, along with higher problematic social media use. Additionally, having a preference for online interaction over face‐to‐face communication has been linked to poorer well‐being among adolescents, including lower self‐esteem (Fioravanti et al., 2012), increased levels of depression and social anxiety (Mýlek et al., 2024), and heightened problematic social media use (van Duin et al., 2021). Since the majority of prior research has utilized cross‐sectional designs, it is unclear whether causal relationships exist between engagement in online interaction and well‐being, although some evidence (Orben, 2020) hints at potential bidirectional relationships.

### A person‐centered approach to social support and online interaction and their links to psychosocial well‐being

The person‐centered approach allows more nuanced structures of social support and online interaction among adolescents in relation to their psychosocial well‐being. Rather than restricting observations to the isolated impacts of different sources of social support and characteristics of online interaction on well‐being, this approach seeks to identify subpopulations within a sample based on selected variables and is frequently employed to examine differences between these subpopulations in terms of different outcomes, including well‐being (Howard & Hoffman, 2018). Importantly, person‐centered research challenges the assumption of population homogeneity in terms of how the studied variables affect each other (Laursen & Hoff, 2006). Moreover, when it comes to testing the existence of subgroups defined by three or more variables, person‐centered approaches tend to be more robust than moderation analyses (or interactions) (Dahling et al., 2017; Morin et al., 2011), which are frequently employed in the variable‐centered approach to test subgroup differences. The complexity of multiway interaction testing inherent in the variable‐centered approach can pose challenges in detecting and interpreting these interactions meaningfully (Meyer & Morin, 2016; Pastor et al., 2007). Consequently, while the person‐centered approach does not replace the variable‐centered approach, it offers a complementary perspective that seems particularly adept at examining the configurations of adolescents' social support and online interaction.

In previous person‐centered studies on social support among adolescents, mostly support offered from and/or perceived from offline social interactions has been studied. The number of profiles—that is, subgroups—identified has varied from three to six, encompassing both convergent profiles (similar levels of social support from all sources) and mixed profiles (mixed levels of support from different sources). The proportion of adolescents falling into the “convergent” profiles with low, moderate, or high levels of support has ranged from 31% to 95% (Chan et al., 2022; Ciarrochi et al., 2017; Jager, 2011; Scholte et al., 2001; Shin & Chang, 2022; Ulmanen, Soini, Pietarinen, & Pyhältö, 2022; Ulmanen, Soini, Pietarinen, Pyhältö, & Rautanen, 2022). Although inconsistencies in the identified mixed patterns exist, several studies have identified profiles characterized by high support from parents or family and low support from friends, or vice versa (Chan et al., 2022; Ciarrochi et al., 2017; Jager, 2011; Scholte et al., 2001; Ulmanen, Soini, Pietarinen, & Pyhältö, 2022; Ulmanen, Soini, Pietarinen, Pyhältö, & Rautanen, 2022).

Convergent profiles obtained from the previous studies on offline support generally support an additive model of improved outcomes, indicating that perceiving higher support from multiple networks is associated with better mental health and academic functioning (Chan et al., 2022; Scholte et al., 2001; Shin & Chang, 2022; Ulmanen, Soini, Pietarinen, & Pyhältö, 2022; Ulmanen, Soini, Pietarinen, Pyhältö, & Rautanen, 2022). However, Chan et al. (2022) observed that strong peer and school support may partially offset the negative effects of low family support, as adolescents who perceived high peer and school support, along with low family support, reported similar levels of self‐efficacy as those with moderate support from multiple networks. Additionally, in the same study, those who perceived minimal support from their family reported higher emotional distress than those who perceived low support from three sources (family, peers, and school), underscoring the important role of family support for adolescents' well‐being. In a study by Ciarrochi et al. (2017), highly parent‐ and peer‐supported 11th graders reported levels of well‐being (e.g., positive mental health) similar to those perceiving high support from parents, peers, and teachers, suggesting that teacher support may not bring additional benefit to well‐supported adolescents. In contrast, a study by Ulmanen, Soini, Pietarinen, Pyhältö, and Rautanen (2022) emphasized the role of teacher support, indicating that despite strong peer and parental support, adolescents reporting low teacher support experienced study burnout symptoms at a similar or even higher level than those perceiving low support from all three sources measured.

Despite the limited application of person‐centered methodologies in investigating both online and offline social interactions, Pan et al. (2023) discovered that offline social capital—the array of material and nonmaterial resources individuals draw from their network of relationships—holds greater significance for adolescent mental health compared with its online counterpart. They specifically observed that adolescents who reported high offline social capital coupled with low online social capital experienced the lowest levels of internalizing symptoms.

It is worth noting that the number of support sources, measures, and analytical approaches (e.g., cluster analysis, mixture models) has varied in previous studies. However, it is evident that “convergence” in relationship quality and online interaction behaviors is not always the case, underscoring the need for further research to explore the heterogeneity in adolescents' support patterns. To date, no study has employed a person‐centered approach to examine how online interaction with specific partners and the prioritization of online interaction over face‐to‐face interactions may alter the links between patterns of social support and psychosocial well‐being, including problematic social media use.

To conclude, online interactions may not encompass the depth found in traditional interpersonal exchanges; yet some individuals may find greater confidence in digital interaction forms. Nevertheless, much of the prior research has not adequately distinguished between different types of online interaction partners, and it is anticipated that individual variations may influence the potential health benefits or detriments stemming from both social support and online interactions. In addition, most previous studies have focused on health outcomes, such as internalizing problems (e.g., depression, anxiety) and psychosocial functioning (e.g., school adjustment, life satisfaction), leaving a gap in research on how social support is linked to other aspects of adolescents' functioning, such as problematic social media use. To address these gaps, this study proposes a complementary view of the structure of offline social support and online interaction, along with their potential links to psychosocial well‐being, by combining variable‐centered and person‐centered methodologies. The data employed in this study were collected across five Nordic countries, which, despite their shared organizational structures within public health systems, still face significant challenges concerning health‐related social inequalities (Fosse & Helgesen, 2019).

### Research aims

Using a variable‐centered approach, this study aimed to (1) examine perceived social support offline from four sources (family, peers, teachers, and classmates), preference for online interaction, and intensity of online interaction as predictors of psychosocial well‐being (psychosomatic complaints and problematic social media use). Additionally, using a person‐centered approach, the study aimed to (2) identify subgroups of adolescents based on their perceptions of social support offline and self‐reported online interaction and examine differences in psychosocial well‐being between the identified profiles.

## METHODS

### Sample

This study is based on cross‐sectional data from the Health Behaviour in School‐aged Children (HBSC) study for the survey year 2017/2018. The sample included 22,384 adolescents in the fifth, seventh, and ninth grades (11‐, 13‐, and 15‐year‐olds) from five countries: Denmark (*n* = 3640), Finland (*n* = 3470), Iceland (*n* = 7144), Norway (*n* = 3842), and Sweden (*n* = 4288). The study followed a standardized protocol (Inchley et al., 2018) for sampling, survey instrument, and data collection. This protocol includes the back‐translation of questions into the source language (English) for comparison against the originals, ensuring the integrity of the survey content. Each country used cluster sampling of classes or schools at a national level to obtain a representative sample. Participants filled in a survey with paper and pen (Sweden) or online (Denmark, Finland, Iceland, and Norway) voluntarily and anonymously during school hours, with no personal identification information collected. Schools also notified parents or guardians of the study and offered them the opportunity to opt out if they preferred that their children did not participate.

### Ethical approval

The HBSC study was conducted according to the guidelines of the Declaration of Helsinki. In Denmark, the study was registered and declared by the Research & Innovation Organisation, University of Southern Denmark. In Finland, the study was approved by the Ethical Committee of the University of Jyväskylä. In Iceland, the study was approved by the Data Protection Authority and the University of Iceland Ethics Committee. In Norway, the Privacy Ombudsman at the Norwegian Social Science Data Services confirmed that the study complied with privacy and confidentiality requirements and no ethical clearance was needed as decided by the Norwegian Western Regional Ethical Committee. In Sweden, no formal approval from an ethical review board was required for this type of study.

### Measures

*Family support* and *peer support* were measured using the Multidimensional Scale of Perceived Social Support (Zimet et al., 1988), which has demonstrated validity (Başol, 2008) and metric invariance across countries (Bi et al., 2021). The scale comprised four items for family (“My family really try to help me,” “I get the emotional help and support I need from my family,” “I can talk about my problems with my family,” and “My family is willing to help me make decisions”) and four items for peers (“My friends really try to help me,” “I can count on my friends when things go wrong,” “I have friends with whom I can share my joys and sorrows,” and “I can talk about my problems with my friends”). In Denmark, responses for family support were measured using a 5‐point Likert scale (from “Very strongly disagree” to “Very strongly agree”), whereas peer support was measured on a 7‐point Likert scale (from “Very strongly disagree” to “Very strongly agree”). In the other four countries, responses were given on a 7‐point Likert scale (from “Very strongly disagree” to “Very strongly agree”) for both scales. For the scales to correspond between countries, the 5‐point scale for the family support items used in Denmark was converted to a 7‐point scale using a linear transformation formula (*Y* = (*B*–*A*) * (*x*–*a*)/(*b*–*a*) + *A*), where (*A*) is the new minimum (7‐point scale), (*B*) is the new maximum (7‐point scale), (a) is the old minimum (5‐point scale), (b) is the old maximum (5‐point scale), and (*x*) is the old value (5‐point scale). This transformation yielded the following formula: *Y* = 1.5 * *x* − 0.5. Next, the items were computed into a mean score (Cronbach's *α* = .97 for family support, Cronbach's *α* = .95 for peer support). Participants with two or more missing items per scale were excluded.

*Teacher support* and *classmate support* were assessed using the Teacher and Classmate Support Scale (Torsheim et al., 2000), a tool that has shown both validity (Torsheim et al., 2012) and metric invariance across different countries (Bi et al., 2021). The scale included three items for teacher support (“I feel that my teachers care about me as a person,” “I feel that my teachers accept me as I am,” and “I trust my teachers a lot”) and three items for classmate support (“The students in my class enjoy being together,” “Most of the students in my class are kind and helpful,” and “Other students accept me as I am”). Responses were given on a 5‐point Likert scale (from “Strongly agree” to “Strongly disagree”). For both scales, items were reversed and computed into a mean score (Cronbach's *α* = .88 for teacher support, Cronbach's *α* = .82 for classmate support). Participants with one or more missing items were excluded.

*Preference for online interaction* was measured using three items adapted from the perceived depth of online communication developed by Peter and Valkenburg (2006). The following statements were presented: “On the Internet, I talk more easily about my secrets/inner feelings/concerns, than in a face‐to‐face encounter.” Adolescents responded on a 5‐point Likert scale (from “Strongly disagree” to “Strongly agree”). Items were computed into a mean score (Cronbach's *α* = .93). Participants with one or more missing items were excluded.

*Intensity of online interaction* was measured using four items adapted from the EU Kids Online survey (Mascheroni & Ólafsson, 2014) with the following introduction: “The next questions are about “online contact” and “online communication”. When we use these terms, we are referring to the exchange of text messages, emoticons, and photo, video, or audio messages through instant messaging platforms (e.g., Snapchat, Instagram, and WhatsApp) or email.” Next, they were asked how often they had online contact with “Close friend(s),” “Friends from a larger friend group,” “Friends/people that you got to know through the internet,” and “Other people (e.g., parents, siblings, classmates, teachers).” Response options were “Does not concern me/I don't know,” “Never or hardly ever,” “At least every week,” “Daily or almost daily,” “Several times a day,” and “Almost all the time.” First, the first two response options were combined because of skewed distributions and because their content was assumed to be fairly equivalent. Notably, for the item concerning “Friends/people that you got to know through the Internet,” over half of the participants selected the first response option, reinforcing the necessity of retaining this category. Additionally, regression analyses revealed that the online interaction items, using their original scale, exhibited a predominantly linear relationship with other key variables. Next, three of the items (online contact with “Close friend(s),” “Friends from a larger friend group,” and “Other people [e.g., parents, siblings, classmates, teachers]”) were computed into a mean score (range: 1–5, Cronbach's *α* = .75), which indicated the level of online interaction adolescents had with their “offline contacts.” Participants with one or more missing items were excluded. The single item “Friends/people that you got to know through the Internet” was used as a continuous variable (range: 1–5) to indicate online interaction with “online contacts.”

*Psychosomatic complaints* were assessed using the HBSC Symptom Checklist (Haugland & Wold, 2001), a validated measure of psychosomatic symptoms (Haugland & Wold, 2001; Ravens‐Sieberer et al., 2010) that has also demonstrated metric invariance across countries (Heinz et al., 2022). Adolescents were asked to indicate how often they have experienced the following symptoms over the last 6 months: headache, stomach‐ache, backache, feeling dizzy, feeling low, irritability or bad mood, feeling nervous, and difficulties in getting to sleep, with five response options (“About every day,” “More than once a week,” “About every week,” “About every month,” and “Rarely or never”). The items were reversed and computed into a mean score (range: 1–5, Cronbach's *α* = .84). Participants with three or more missing items were excluded.

*Problematic social media use* was measured using the nine‐item Social Media Disorder Scale (Van den Eijnden et al., 2016), which has demonstrated validity (Boer et al., 2020) and scalar invariance across countries (Boer et al., 2022). Adolescents reported whether they, during the past year, regularly could not think of anything else but the moment that they would be able to use social media again, regularly felt dissatisfied because they wanted to spend more time on social media, often felt bad when they could not use social media, tried to spend less time on social media but failed, regularly neglected other activities (e.g., hobbies, sports) because they wanted to use social media, regularly had arguments with others because of their social media use, regularly lied to parents or friends about the amount of time they spend on social media, often used social media to escape from negative feelings, and had serious conflicts with parents or siblings because of their social media use. Response options were “Yes” and “No.” All yes responses were summed (range: 0–9) and used as a continuous score (Cronbach's *α* = .80). Participants with three or more missing items were excluded.

#### Sociodemographic characteristics

The following sociodemographic characteristics were considered: *gender* (boys and girls), *grade* (5, 7, and 9), *family structure* (lives in a nuclear family, single‐parent family, step‐family, or in foster care or children's home), *family affluence*, *immigrant background* (first‐generation immigrant = born abroad, second‐generation immigrant = born in the survey country and one or both parents born abroad, and native/non‐immigrant background = the respondent and parent(s) were born in the survey country), and *country*.

*Family affluence* was measured using the six‐item HBSC Family Affluence Scale III (Torsheim et al., 2015): “Does your family own a car, van, or truck?” (“No,” “Yes, one,” “Yes, two or more”), “Do you have your own bedroom for yourself?” (“No,” “Yes”), “How many computers does your family own (including laptops and tablets, not including game consoles and smartphones)?” (“None,” “One,” “Two,” “More than two”), “How many bathrooms (room with a bath/shower or both) are in your home?” (“None,” “One,” “Two,” “More than two”), “Does your family have a dishwasher at home?” (“No,” “Yes”), “How many times did you and your family travel abroad for a holiday/vacation last year?” (“Not at all,” “Once,” “Twice,” “More than twice”). A sum score (range: 0–13) was calculated from these items. Participants with two or more missing items were excluded.

### Measurement invariance

Prior to the analyses, we evaluated measurement invariance (MI) across countries to confirm that the factor structure of the previously untested online interaction variables was consistent and that the measures were interpreted similarly by participants from different countries. We used multigroup confirmatory factor analysis (CFA) with the “lavaan” and “semTools” packages in R to evaluate the preference for and intensity of online interaction.

As three indicators per factor are needed to identify common factors and avoid empirical underidentification (Brown, 2015; Floyd & Widaman, 1995), we excluded the single item indicating online interaction with “online contacts” from the CFA. Thus, we estimated a baseline model that included two correlated factors: preference for online interaction and intensity of online interaction with “offline contacts,” each loaded by three indicators.

We employed Weighted Least Squares Mean and Variance‐adjusted (WLSMV) estimation in all CFA analyses to account for the ordinal nature of the item scores. We tested four increasingly restrictive levels of MI: (1) configural invariance (free intercepts and loadings across countries), (2) threshold invariance (free intercepts and constrained thresholds), (3) metric invariance (free intercepts with constrained loadings and thresholds), and (4) scalar invariance (constrained intercepts, loadings, and thresholds) (Svetina et al., 2020; Wu & Estabrook, 2016).

Model fit was evaluated using several goodness‐of‐fit indices: Comparative Fit Index (CFI), Tucker–Lewis Index (TLI), Root Mean Square Error of Approximation (RMSEA), and Standardized Root Mean Square Residual (SRMR) (Chen, 2007; Fischer & Karl, 2019; Svetina et al., 2020). For testing configural invariance, models with CFI >0.90, TLI >0.90, RMSEA <0.08, and SRMR <0.08 were considered to have acceptable fit (Hu & Bentler, 1999; van de Schoot et al., 2012). For testing metric invariance, a change (Δ) of < −0.010 in CFI, <0.015 in RMSEA, and <0.030 in SRMR indicated invariance (Bluemke et al., 2023; Chen, 2007). For testing scalar invariance, a change of < −0.010 in CFI, <0.015 in RMSEA, and <0.010 in SRMR indicated invariance (Bluemke et al., 2023; Chen, 2007).

According to the fit indices presented in Table S1, our study demonstrated configural, threshold, and metric invariance. The fit indices for the configural invariance model were excellent (scaled CFI = 0.999, TLI = 0.999, RMSEA = 0.041, and SRMR = 0.020). Threshold invariance was also supported with minimal changes in fit indices (ΔCFI <0.001, ΔRMSEA = 0.001, and ΔSRMR <0.001). Metric invariance was confirmed with changes in fit indices well within acceptable limits (ΔCFI = 0.001, ΔRMSEA = 0.002, and ΔSRMR = 0.004). Scalar invariance was largely supported, as indicated by ΔCFI = 0.002 and ΔSRMR = 0.001. However, the change in RMSEA (ΔRMSEA = 0.017) was considered slightly too large, suggesting some limitations in achieving full scalar invariance.

### Statistical analysis

We computed descriptive statistics using the chi‐square test, analysis of variance (ANOVA), post hoc ANOVA with Bonferroni correction, and Pearson correlation. We addressed missing data through listwise deletion. The distribution of missingness varied across key variables: family support (3.1%), peer support (4.5%), teacher support (5.6%), classmate support (4.8%), preference for online interaction (10.9%), intensity of online interaction with “offline contacts” (5.5%) and “online contacts” (7.5%), psychosomatic complaints (2.2%), and problematic social media use (11.9%).

To assess sociodemographic differences between the groups with missing data and those with complete data, we employed independent *t*‐tests and chi‐square tests. We calculated Cohen's *d* to determine the effect size of mean differences for the continuous variable, while Cramer's *V* was used to compute effect sizes for categorical variables.

Compared to excluded adolescents, those included in the study were significantly more likely to be female (51.9% vs. 41.4%), in ninth grade (32.4% vs. 22.1%), living in a nuclear family (72.3% vs. 67.7%), and having a non‐immigrant background (77.9% vs. 69.6%). Additionally, included adolescents scored higher on family affluence (*M* = 9.34 vs. 9.05). Geographically, included adolescents were more likely to come from Denmark (16.9% vs. 13.2%), Finland (16.5% vs. 10.5%), and Iceland (32.3% vs. 29.8%), while being less likely to be from Norway (15.5% vs. 25.5%) and Sweden (18.8% vs. 20.9%) compared to those excluded. Following Cohen's criteria for effect sizes (Cohen, 1988, 1992), all effect sizes for the sociodemographic differences between included and excluded adolescents were considered small, with Cohen's *d* = 0.15 and Cramer's *V* ranging from 0.06 to 0.12.

For the variable‐centered analysis, we estimated linear mixed models using restricted maximum likelihood (REML) estimation to examine how different sources of perceived social support (family, peers, teachers, and classmates), preference for online interaction, and intensity of online interaction were associated with psychosocial well‐being. The outcomes included psychosomatic complaints and problematic social media use, which were analyzed separately. We controlled for sociodemographic characteristics in these models. No multicollinearity was detected among the independent variables, as indicated by variance inflation factors <2. To account for the nested structure of the data, with pupils nested within schools, we treated “school” as a random effect. We performed two adjusted models: the first included all sociodemographic characteristics as covariates, and the second encompassed all independent variables. Finland was set as the reference category because, according to most indicators, adolescents in this country reported the lowest levels of social support and the highest intensity of online interaction.

For the person‐centered analysis, we conducted latent profile analysis (LPA) using Gaussian finite mixture modeling for seven indicators (comprising social support and online interaction variables). First, we transformed the raw score of each indicator into a z‐score for the analysis; a z‐score of 0 represents the overall sample mean. We utilized the R package tidyLPA (Rosenberg et al., 2018) to conduct the LPA. This package provides four specific types of parameterizations (Models 1, 2, 3, and 6), each with different constraints on the variance (varying or equal) and covariance (varying, zero, or equal) of the profiles. We compared different numbers of profiles (from 1 to 10) and relied on several statistical indicators to determine the best‐fitting model: Akaike's information criterion (AIC; Akaike, 1987), Bayesian information criterion (BIC; Schwarz, 1978), entropy (Celeux & Soromenho, 1996), and the bootstrap likelihood ratio test (BLRT; McLachlan, 1987). Lower values of AIC and BIC suggest a better model fit. Entropy measures classification accuracy, indicating to what extent individuals are correctly classified within latent profiles; it ranges from 0 to 1, with values preferably at or >.80 indicating high accuracy (Clark & Muthén, 2009). A small *p*‐value for the BLRT signifies that the *K*_0_‐class model fits the data significantly better than the *K*_−1_‐class model (Tein et al., 2013). Nonetheless, it should be noted that larger sample sizes can lead to significant BLRT values even when practical significance is low (Grimm et al., 2021). As such, we selected the optimal number of classes by identifying the point where the slope of improvements flattened (Morin et al., 2016). We also took into account theoretical interpretability, the discrimination among profile indicators, and the size of the profiles (Woo et al., 2018). Given that our sample size was large and that simulation studies have indicated that smaller subgroups with rare patterns are more easily identified in larger samples (Morgan, 2015), we deemed profiles with <5% of the total sample size acceptable if the minimum probability was >.80. Finally, we visually inspected the profile solutions to select the final model.

After selecting the final profile solution, adolescents were assigned to their most likely profile. Uncertainty of class membership was incorporated into subsequent analyses by using each individual's posterior probabilities as weights (Clark & Muthén, 2009; Kamata et al., 2018). These probabilities represent the likelihood of class membership for each observation after the model has been fit, with values closer to 1.0 indicating a higher probability of membership in a specific profile. We used the chi‐square test, ANOVA, and post hoc ANOVA with Bonferroni correction to compare differences in profile indicators and sociodemographic characteristics between profiles. To examine associations between the emerging profiles and psychosocial well‐being, we used linear mixed models with REML estimation. Additionally, to examine the proportion of adolescents classified as problematic social media users (i.e., a score of 6 or more on a scale from 0 to 9) across the different profiles, generalized linear mixed models with binary logistic regression were conducted, treating problematic social media use as a binary outcome. We controlled for sociodemographic characteristics, and “school” was included as a random effect.

We conducted descriptive statistics and linear mixed models using SPSS version 29. For the LPA, we utilized R 4.3.0. We conducted sensitivity analyses using multiple imputation, which showed no significant differences in social support, online interaction, and psychosocial well‐being between the imputed and original values, and the findings were highly similar to those obtained using listwise deletion.

## RESULTS

### Descriptive statistics

Table 1 presents sociodemographic characteristics, social support, online interaction, and psychosocial well‐being per country. The overall means for the key variables were as follows: family support (*M* = 5.90, range: 1–7), peer support (*M* = 5.56, range: 1–7), teacher support (*M* = 4.02, range: 1–5), classmate support (*M* = 3.99, range: 1–5), preference for online interaction (*M* = 2.49, range: 1–5), intensity of online interaction with “offline contacts” (*M* = 2.97, range: 1–5) and “online contacts” (*M* = 1.91, range: 1–5), psychosomatic complaints (*M* = 2.15, range: 1–5), and problematic social media use (*M* = 1.59, range: 0–9). Overall, the lowest mean levels of social support were reported in Finland and Iceland. The lowest levels of online interaction with “offline contacts” were reported in Iceland, whereas the lowest levels of online interaction with “online contacts” were reported in Denmark, Iceland, and Norway. The countries with the highest mean levels of psychosomatic complaints were Sweden, Finland, and Iceland, while Finland and Norway reported the highest mean levels of problematic social media use. Correlations between the variables of social support, online interaction, and psychosocial well‐being are presented in Table 2.

**TABLE 1:** Sample characteristics per country.

	All (*n* = 19,717–22,384)	Denmark (*n* = 3228–3640)	Finland (*n* = 3190–3470)	Iceland (*n* = 6360–7144)	Norway (*n* = 3191–3842)	Sweden (*n* = 3748–4288)	Overall	*p*‐value	Multiple comparison/group differences
	% (*n*)/*M* (*SD*)	% (n)/M (SD)	% (*n*)/*M* (*SD*)	% (*n*)/*M* (*SD*)	% (*n*)/*M* (*SD*)	% (*n*)/*M* (*SD*)	*χ* ^2^/*F*		
Sociodemographic characteristics									
Gender, female (vs. male)	50.19 (11111)	49.97 (1819)	49.88 (1712)	49.77 (3496)	51.47 (1972)	50.17 (2112)	3.22	.522	No significant differences
Mean age	13.57 (1.66)	13.52 (1.68)	13.97 (1.62)	13.60 (1.63)	13.13 (1.64)	13.64 (1.64)	121.03	<.001	Denmark^a^, Finland^b^, Iceland^ac^, Norway^d^, Sweden^c^
Grade							669.56	<.001	
Fifth	35.74 (8000)	40.69 (1481)	29.97 (1040)	32.95 (2354)	50.60 (1944)	27.54 (1181)			Denmark^a^, Finland^b^, Iceland^c^, Norway^d^, Sweden^b^
Seventh	33.60 (7520)	33.60 (1223)	36.80 (1277)	35.64 (2546)	26.60 (1022)	33.86 (1452)			Denmark^a^, Finland^b^, Iceland^ab^, Norway^c^, Sweden^ab^
Ninth	30.66 (6864)	25.71 (936)	33.23 (1153)	31.41 (2244)	22.80 (876)	38.60 (1655)			Denmark^a^, Finland^b^, Iceland^b^, Norway^c^, Sweden^d^
Family affluence	9.29 (1.92)	9.54 (1.92)	8.80 (1.93)	9.16 (1.85)	9.80 (1.78)	9.27 (2.03)	148.32	<.001	Denmark^a^, Finland^b^, Iceland^c^, Norway^d^, Sweden^e^
Family structure							101.53	<.001	
Nuclear family	71.66 (15087)	72.92 (2550)	74.19 (2498)	70.71 (4828)	71.36 (2262)	70.33 (2949)			Denmark^ab^, Finland^a^, Iceland^b^, Norway^ab^, Sweden^b^
Single‐parent family	16.33 (3438)	15.78 (552)	13.45 (453)	16.07 (1097)	18.20 (577)	18.10 (759)			Denmark^ab^, Finland^a^, Iceland^b^, Norway^ab^, Sweden^b^
Stepfamily	10.92 (2299)	10.32 (361)	11.46 (386)	12.51 (854)	9.05 (287)	9.80 (411)			Denmark^ab^, Finland^ac^, Iceland^c^, Norway^b^, Sweden^ab^
Foster care or children's home	1.10 (231)	1.00 (34)	0.90 (30)	0.70 (49)	1.40 (44)	1.80 (74)			Denmark^ab^, Finland^ab^, Iceland^a^, Norway^bc^, Sweden^c^
Immigrant background							768.01	<.001	
Nonimmigrant	76.57 (16852)	77.53 (2785)	88.77 (3021)	78.36 (5556)	74.74 (2832)	64.28 (2658)			Denmark^a^, Finland^b^, Iceland^a^, Norway^c^, Sweden^d^
First‐generation immigrant	8.67 (1909)	5.99 (215)	4.17 (142)	10.27 (728)	8.47 (321)	12.16 (503)			Denmark^a^, Finland^b^, Iceland^c^, Norway^d^, Sweden^e^
Second‐generation immigrant	14.76 (3248)	16.48 (592)	7.05 (240)	11.37 (806)	16.79 (636)	23.56 (974)			Denmark^a^, Finland^b^, Iceland^c^, Norway^a^, Sweden^d^
Social support and online variables									
Family support	5.90 (1.54)	6.17 (1.01)	5.68 (1.66)	5.63 (1.93)	6.22 (1.10)	6.04 (1.30)	149.50	<.001	Denmark^a^, Finland^b^, Iceland^b^, Norway^a^, Sweden^c^
Peer support	5.56 (1.65)	5.98 (1.35)	5.44 (1.67)	5.29 (1.88)	5.70 (1.44)	5.65 (1.50)	116.49	<.001	Denmark^a^, Finland^b^, Iceland^c^, Norway^d^, Sweden^d^
Teacher support	4.02 (0.89)	4.01 (0.87)	3.85 (0.94)	4.02 (0.87)	4.13 (0.89)	4.10 (0.86)	54.24	<.001	Denmark^a^, Finland^b^, Iceland^a^, Norway^c^, Sweden^c^
Classmate support	3.99 (0.78)	4.03 (0.76)	3.90 (0.78)	3.96 (0.78)	4.18 (0.78)	3.92 (0.78)	71.94	<.001	Denmark^a^, Finland^b^, Iceland^c^, Norway^d^, Sweden^b^
Preference for online interaction	2.49 (1.21)	2.47 (1.10)	2.54 (1.18)	2.38 (1.24)	2.46 (1.23)	2.68 (1.24)	38.23	<.001	Denmark^a^, Finland^a^, Iceland^b^, Norway^a^, Sweden^c^
Intensity of online interaction with “offline contacts”	2.97 (1.10)	3.00 (1.07)	3.03 (0.95)	2.89 (1.13)	3.00 (1.12)	3.01 (1.17)	13.40	<.001	Denmark^a^, Finland^a^, Iceland^b^, Norway^a^, Sweden^a^
Intensity of online interaction with “online contacts”	1.91 (1.32)	1.88 (1.30)	2.07 (1.27)	1.85 (1.31)	1.82 (1.29)	2.00 (1.39)	24.45	<.001	Denmark^a^, Finland^b^, Iceland^a^, Norway^a^, Sweden^b^
Psychosomatic complaints	2.15 (0.85)	1.97 (0.75)	2.24 (0.83)	2.20 (0.92)	1.94 (0.76)	2.32 (0.84)	157.88	<.001	Denmark^a^, Finland^b^, Iceland^b^, Norway^a^, Sweden^c^
Problematic social media use	1.59 (2.09)	1.43 (1.82)	1.96 (2.35)	1.28 (1.96)	1.91 (2.38)	1.64 (1.94)	85.01	<.001	Denmark^a^, Finland^b^, Iceland^c^, Norway^d^, Sweden^e^

*Note*: Chi‐square test for percentage comparison and post hoc analysis of variance for mean comparison (two‐tailed). Scores ranged from 10 to 16 for age, 0 to 13 for family affluence, 1 to 7 for family and peer support, 1 to 5 for teacher and classmate support, 1 to 5 for preference for online interaction, 1 to 5 for intensity of online interaction, 1 to 5 for psychosomatic complaints, and 0 to 9 for problematic social media use. Countries with different subscript letters had significantly different (*p* < .05) column proportions when Bonferroni‐corrected multiple comparisons were used.

**TABLE 2:** Correlations of social support, online interaction, and psychosocial well‐being (*n* = 19,263–21,694).

	1	2	3	4	5	6	7	8
1 Family support^a^	–							
2 Peer support^a^	.59*	–						
3 Teacher support^a^	.25*	.20*	–					
4 Classmate support^a^	.20*	.25*	.44*	–				
5 Preference for online interaction^b^	−.13*	−.07*	−.13*	−.08*	–			
6 Intensity of online interaction with “offline contacts”^c^	.04*	.17*	−.04*	.08*	.17*	–		
7 Intensity of online interaction with “online contacts”^c^	−.10*	−.01	−.14*	−.05*	.28*	.44*	–	
8 Psychosomatic complaints^d^	−.26*	−.18*	−.29*	−.31*	.18*	.05*	.15*	–
9 Problematic social media use^e^	−.14*	−.06*	−.16*	−.12*	.23*	.16*	.24*	.27*

*Note*: Pearson product–moment correlation.

^a^ Higher values indicate higher support.

^b^ Higher values indicate higher preference for online interaction.

^c^ Higher values indicate higher intensity of online interaction.

^d^ Higher values indicate more frequent psychosomatic complaints.

^e^ Higher values indicate higher problematic social media use.

* *p* < .001.

### Variable‐centered results

The results of the regression models suggested fairly similar associations between predictors (social support and online interaction) and the two psychosocial well‐being outcomes studied (psychosomatic complaints, and problematic social media use) (Table 3). Thus, higher levels of support from family, peers, teachers, and classmates were associated with lower levels of psychosomatic complaints in the fully adjusted model (adjusted b). In contrast, a greater preference for online interaction and more intense interaction with “online contacts” were associated with higher levels of both psychosomatic complaints and problematic social media use. The associations observed between social support and online interaction variables and problematic social media use were akin, although a bit weaker, with two exceptions: Peer support was not linked to problematic social media use, while more frequent online interaction with “offline contacts” was associated with increased problematic social media use but was not related to psychosomatic complaints. Associations between sociodemographic characteristics and psychosocial well‐being are presented in Table S2.

**TABLE 3:** Results from the mixed‐effects linear regression showing associations between social support, online interaction, and psychosocial well‐being.

		Psychosomatic complaints (*n* = 17,568–20,070)				Problematic social media use (*n* = 17,401–18,633)			
		*B* (95% CI)	*β*	*p*‐Value	Marginal pseudo *R* ^2^	*B* (95% CI)	*β*	*p*‐Value	Marginal pseudo *R* ^2^
Family support	Adjusted^a^	**−0.11 (−0.12, −0.11)**	**−0.21**	**<.001**	.146	**−0.17 (−0.19, −0.15)**	**−0.13**	**<.001**	.060
	Adjusted^b^	**−0.06 (−0.07, −0.05)**	**−0.11**	**<.001**	.237	**−0.11 (−0.13, −0.09)**	**−0.08**	**<.001**	.139
Peer support	Adjusted^a^	**−0.09 (−0.10, −0.08)**	**−0.18**	**<.001**	.134	**−0.08 (−0.10, −0.07)**	**−0.07**	**<.001**	.048
	Adjusted^b^	**−0.02 (−0.03, −0.01)**	**−0.03**	**<.001**		0.01 (−0.01, 0.03)	0.01	.378	
Teacher support	Adjusted^a^	**−0.25 (−0.26, −0.24)**	**−0.26**	**<.001**	.166	**−0.31 (−0.35, −0.28)**	**−0.13**	**<.001**	.060
	Adjusted^b^	**−0.14 (−0.16, −0.13)**	**−0.15**	**<.001**		**−0.16 (−0.20, −0.12)**	**−0.07**	**<.001**	
Classmate support	Adjusted^a^	**−0.28 (−0.30, −0.27)**	**−0.26**	**<.001**	.170	**−0.27 (−0.31, −0.23)**	**−0.10**	**<.001**	.054
	Adjusted^b^	**−0.18 (−0.20, −0.16)**	**−0.16**	**<.001**		**−0.14 (−0.18, −0.09)**	**−0.05**	**<.001**	
Preference for online interaction	Adjusted^a^	**0.11 (0.10, 0.12)**	**0.16**	**<.001**	.132	**0.36 (0.34, 0.38)**	**0.21**	**<.001**	.086
	Adjusted^b^	**0.07 (0.06, 0.08)**	**0.10**	**<.001**		**0.25 (0.23, 0.28)**	**0.15**	**<.001**	
Intensity of online interaction with “offline contacts”	Adjusted^a^	0.01 (−0.01, 0.02)	0.01	.335	.106	**0.27 (0.24, 0.30)**	**0.14**	**<.001**	.060
	Adjusted^b^	0.00 (−0.01, 0.01)	0.00	.898		**0.14 (0.11, 0.17)**	**0.07**	**<.001**	
Intensity of online interaction with “online contacts”	Adjusted^a^	**0.08 (0.07, 0.09)**	**0.13**	**<.001**	.120	**0.35 (0.33, 0.38)**	**0.22**	**<.001**	.092
	Adjusted^b^	**0.05 (0.04, 0.06)**	**0.08**	**<.001**		**0.24 (0.21, 0.26)**	**0.15**	**<.001**	

*Note*: *B*, unstandardized regression coefficient; CI, confidence interval; *β*, standardized regression coefficient. Marginal *R* ^2^ represents the variance explained by independent variables using *R* ^2^ statistics of Nakagawa and Schielzeth (2013). Bold values denote statistical significance.

^a^ Adjusted for sociodemographic characteristics (gender, grade, family affluence, family structure, immigrant background, and country).

^b^ Adjusted for sociodemographic characteristics, social support, and online variables (preference for online interaction, and intensity of online interaction).

### Person‐centered results

#### Latent profile analysis

The fit indices for the LPA based on seven indicators (social support and online interaction variables) are presented in Table 4. Among the four models tested, Model 1, which assumed equal variances and covariances fixed to zero, provided the best fit indices. The 4‐ and 5‐class solutions fitted the data best. We selected the 5‐class solution as the most optimal based on the subsequently declining AIC and BIC values, higher entropy, and greater interpretability. Additionally, greater decreases in AIC and BIC occurred when comparing the 4‐ and 5‐class solutions rather than the 3‐ and 4‐class solutions, indicating that the 5‐class solution was a superior fit. We present the examined plots for different class solutions (Models 1 and 3) in Figure S1.

**TABLE 4:** Fit indices for latent profile analysis results with Models 1, 2, 3, and 6.

Model	Number of profiles	AIC	BIC	Entropy	Minimum average probability	Smallest *n* (%)	BLRT (*p*)
Model 1 (equal variance; covariance fixed to zero)	1	370684.64	370794.31	1.00	1.00	1.00	−
	2	353015.22	353187.57	0.95	0.94	0.13	.01
	3	342669.26	342904.28	0.92	0.94	0.10	.01
	4	337698.06	337995.75	0.87	0.80	0.07	.01
	**5**	**332258.90**	**332619.27**	**0.92**	**0.84**	**0.04**	.**01**
	6	330241.94	330664.98	0.90	0.69	0.04	.01
	7	329249.84	329735.55	0.83	0.57	0.05	.01
	8	328949.36	329497.75	0.74	0.64	0.05	.01
	9	326895.20	327506.26	0.78	0.66	0.02	.01
	10	326364.42	327038.15	0.75	0.58	0.01	.01
Model 2 (varying variance; covariance fixed to zero)	1	370684.64	370794.31	1.00	1.00	1.00	−
	2–10^a^	–					
Model 3 (equal variance; equal covariance)	1	350228.00	350502.19	1.00	1.00	1.00	−
	2	339259.38	339596.24	0.96	0.99	0.28	.01
	3	338983.87	339383.41	0.65	0.68	0.28	.01
	4	332503.16	332965.37	0.73	0.40	0.07	.01
	5	330460.77	330985.65	0.61	0.53	0.06	.76
	6	330475.23	331062.79	0.61	0.33	0.06	.01
	7^b^	327368.79	328019.02	0.61	0.32	0.05	.01
	8^b^	329320.08	330032.98	0.60	0.00	0.00	.01
	9^b^	326532.28	327307.85	0.59	0.00	0.00	.01
	10^b^	329373.97	330212.22	0.53	0.00	0.00	.01
Model 6 (varying variance; varying covariance)	1	350228.00	350502.19	1.00	1.00	1.00	−
	2–10^a^	−					

*Note*: AIC, Akaike's information criterion; BIC, Bayesian information criterion; BLRT, bootstrap likelihood ratio test; minimum probability: minimum class membership probability within the profile solution. Number of profiles (classes) ranged from 1 to 10. Bold row shows the selected solution.

^a^ Estimation did not converge for these models.

^b^ Model resulted in a warning message.

**FIGURE 1:**
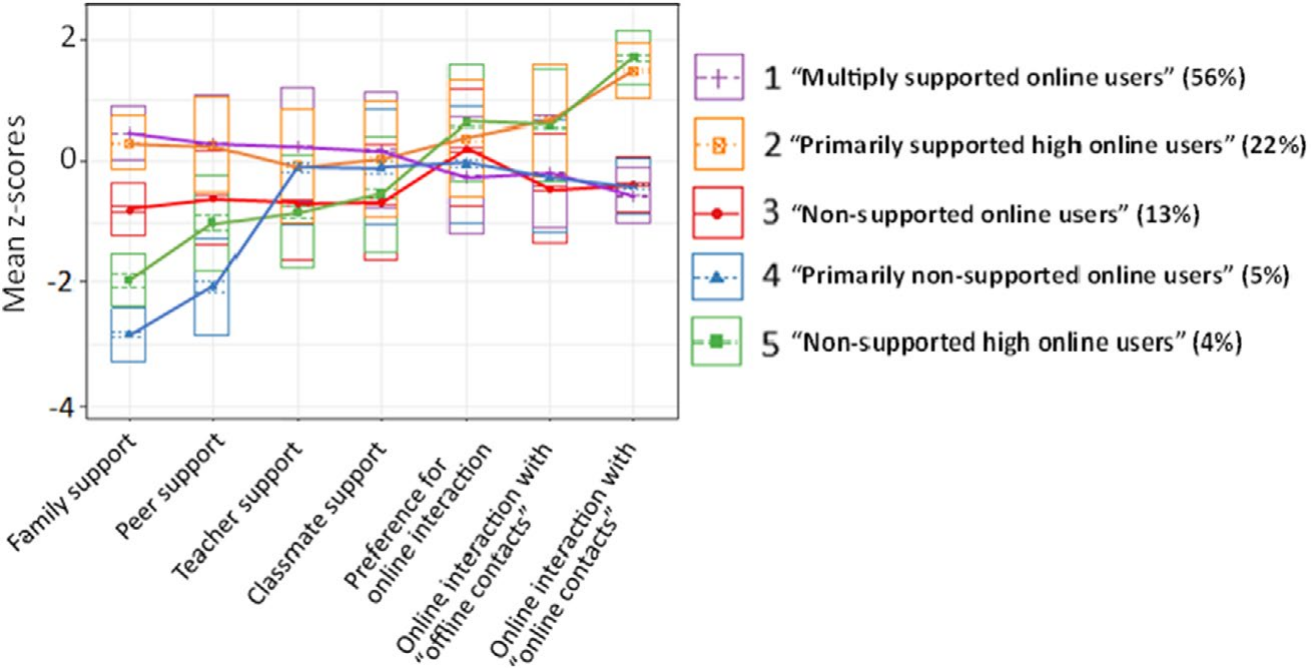
Line graph indicating the 5‐class solution (Model 1) showing standardized mean values on the *y*‐axis and the profile indicators on the *x*‐axis. Bars represent 95% confidence intervals around the standardized value for a given indicator. The data are weighted by the posterior probabilities of the units for the 5‐class solution.

The five profiles derived from the most optimal model (shown in Figure 1 and Table S3) included adolescents reporting: (1) High social support from all sources, low preference for online interaction, and moderate online interaction (“Multiply supported online users” 56%), (2) high social support from family and peers (i.e., their primary group), moderate support from teachers and classmates, high preference for online interaction, and high online interaction (“Primarily supported high online users” 22%), (3) low social support, high preference for online interaction, and moderate online interaction (“Non‐supported online users” 13%), (4) very low family and peer support, moderate teacher and classmate support, and moderate preference for, and intensity of, online interaction (“Primarily non‐supported online users” 5%), (5) low social support, high preference for online interaction, and high online interaction (“Non‐supported high online users” 4%). Those classified as “high online users” typically engaged in online interactions several times a day, while those characterized as more general “online users” engaged in online behaviors daily or almost daily on average.

#### Sociodemographic characteristics of the profiles

Chi‐square tests and one‐way ANOVAs (see Table S3) revealed minimal differences between the profiles in terms of gender and immigrant background. The distribution of girls versus boys across the profiles was fairly even, with the highest proportion (56%) of girls concentrated in the “Non‐supported high online users” profile, while the “Primarily supported high online users” profile included the highest proportion (54%) of boys. In all profiles, the majority (75%–79%) were nonimmigrants, with roughly one tenth (14%–16%) being second‐generation immigrants and slightly fewer than one tenth (7%–10%) being first‐generation immigrants. Moreover, the “Multiply supported online users” profile comprised a higher proportion of fifth graders, adolescents from nuclear families, and individuals from Norway, and fewer adolescents from Finland compared with the other four profiles. Those in the “Multiply supported online users” and “Primarily supported high online users” profiles also reported slightly higher family affluence than those in the remaining three profiles. The proportion of adolescents from Denmark, Norway, and Sweden was the lowest, while that from Iceland was the highest in the “Primarily non‐supported online users” profile compared with the other profiles.

#### Associations between profile membership and psychosocial well‐being

**FIGURE 2:**
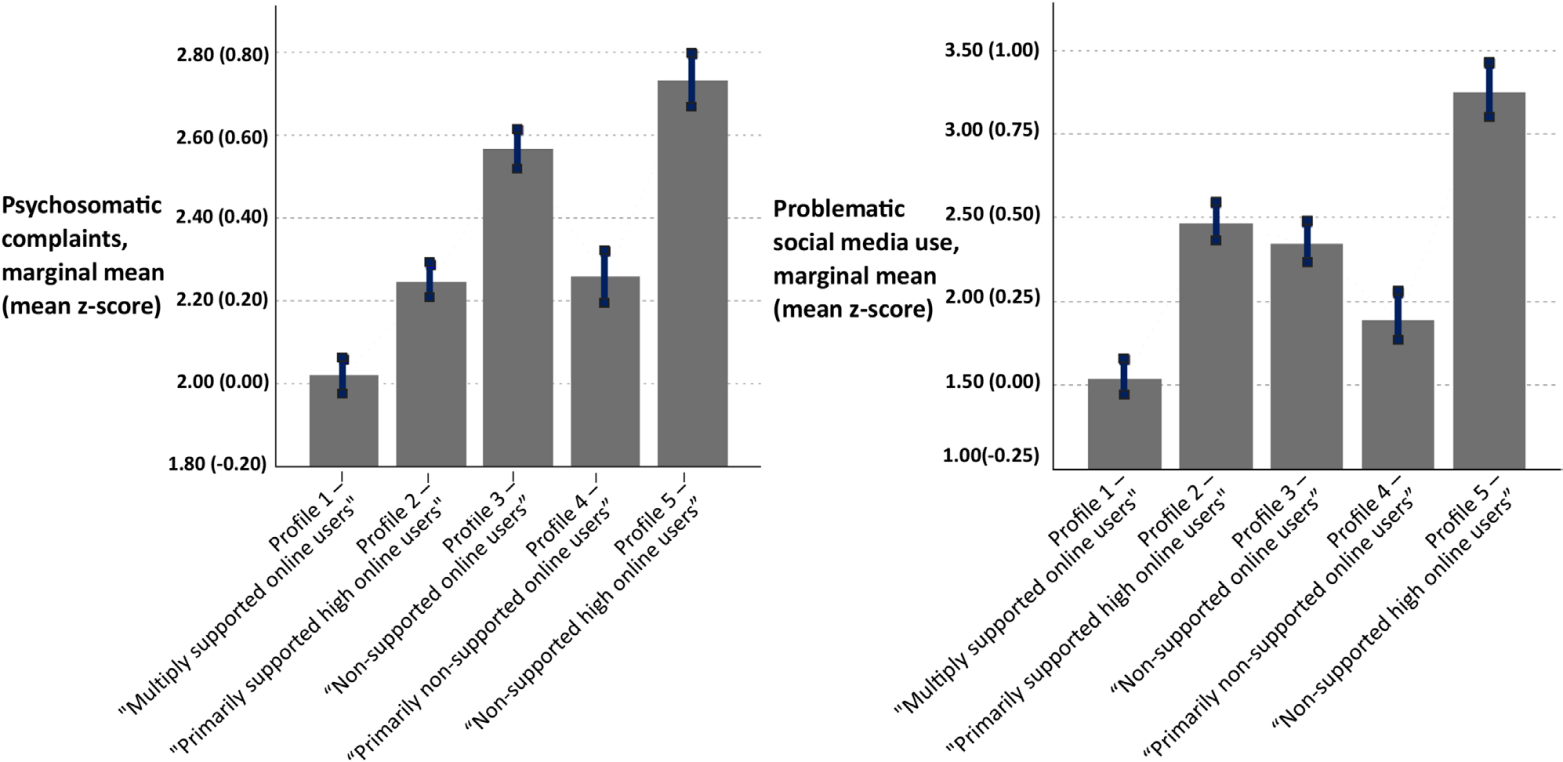
Bar graphs showing marginal means for psychosocial well‐being outcomes across the identified profiles when adjusting for covariates and the nested structure of the data. Bars represent 95% confidence intervals for the mean value of a psychosocial well‐being outcome. Scores ranged from 1 to 5 for psychosomatic complaints and 0 to 9 for problematic social media use. The data are weighted by the posterior probabilities of the units for the 5‐class solution.

Adjusted linear mixed models revealed that adolescents in the “Multiply supported online users” profile reported lower levels of psychosomatic complaints and problematic social media use compared with those in the other profiles (Table 5). As shown in Figure 2, the marginal mean for psychosomatic complaints was highest in the “Non‐supported high online users” profile, followed by the “Non‐supported online users,” “Primarily non‐supported online users,” and the “Primarily supported high online users” profiles. Similarly, the marginal mean for problematic social media use was highest in the “Non‐supported high online users” profile, but it was followed by the “Primarily supported high online users,” “Non‐supported online users,” and the “Primarily non‐supported online users” profiles. When examining the proportion of adolescents classified as problematic social media users (i.e., a score of 6 or more on a scale from 0 to 9) across the different profiles, the “Multiply supported online users” profile and the “Primarily non‐supported online users” profile had the lowest proportions at 4% and 6%, respectively, with no significant difference between them (see Figure S2). In contrast, the “Non‐supported high online users” profile exhibited the highest proportion at 23%, followed by the “Primarily supported high online users” profile at 12%, and the “Non‐supported online users” profile at 11%.

**TABLE 5:** Associations between the identified profiles and psychosocial well‐being.

	Psychosomatic complaints		Problematic social media use	
	*B* (95% CI)	*p*‐Value	*B* (95% CI)	*p*‐Value
Profile (reference Profile 1—Multiply supported online users)				
Profile 2—Primarily supported high online users	**0.23 (0.20, 0.25)**	**<.001**	**0.93 (0.85, 1.00)**	**<.001**
Profile 3—Non‐supported online users	**0.55 (0.51, 0.58)**	**<.001**	**0.80 (0.71, 0.90)**	**<.001**
Profile 4—Primarily non‐supported online users	**0.24 (0.19, 0.29)**	**<.001**	**0.34 (0.21, 0.48)**	**<.001**
Profile 5—Non‐supported high online users	**0.71 (0.65, 0.77)**	**<.001**	**1.71 (1.56, 1.85)**	**<.001**
Marginal pseudo *R* ^2^	.170		.100	

*Note*: Linear mixed‐effects regression models per profile: *B*, unstandardized regression coefficients; CI, confidence interval. Marginal *R* ^2^ represents the variance explained by independent variables using *R* ^2^ statistics of Nakagawa and Schielzeth (2013). The data are weighted by the posterior probabilities of the units for the 5‐class solution. Adjusted for sociodemographic characteristics (gender, grade, family affluence, family structure, immigrant background, and country). Bold values denote statistical significance.

## DISCUSSION

By integrating variable‐centered and person‐centered methodologies, this study presented a complementary view of adolescents' different online interactive behaviors and offline social support structures, along with their potential links to psychosocial well‐being. The study also capitalized on the need to enlarge the scope of the well‐being approach among digital natives to encompass their problematic social media use along with traditionally used indicators of psychosomatic health. Overall, our results consistently underscore the health benefits of securing social support across various networks. However, insights from the person‐centered analysis indicate that robust support from specific sources in adolescents' educational context may partially mitigate the adverse effects of insufficient support elsewhere. Furthermore, this multifaceted approach demonstrates that the relationship between online interaction and psychosocial well‐being is partly dependent on the level of social support available to individuals.

The results from our variable‐centered analysis indicate that adolescents who perceive strong offline social support from various sources, who show a lower preference for online interaction over face‐to‐face interaction, and who engage in less frequent online interaction, generally report better psychosocial well‐being. Notably, the significance of support from teachers and classmates was particularly compelling, as these sources demonstrated a stronger negative association with psychosomatic complaints compared with other sources. This finding is partly consistent with previous evidence from meta‐analyses (Chu et al., 2010; Kim et al., 2018) which observed that teacher support was more strongly linked with adolescents' well‐being than support from parents or peers. Additionally, our analysis revealed that while high levels of peer support were weakly linked to better psychosomatic health, this support seemed to have no apparent relationship to problematic social media use. One potential explanation for this lack of significant connection could be that peers themselves are also prone to problematic social media use. Furthermore, online interaction with online contacts, rather than offline contacts, correlated with increased psychosomatic complaints. Conversely, a high frequency of online interaction, regardless of the interaction partner, was associated with increased problematic social media use. Overall, our variable‐centered findings point to a nuanced differential impact of social dynamics and online interaction on individual well‐being. While the quality of social relationships appears to be more strongly associated with psychosomatic complaints, online interaction behaviors demonstrate a stronger connection to problematic social media use on average.

In the person‐centered analysis, and in line with previous studies on profiles of social support, we identified profiles that exhibited both converging and diverging characteristics. Notably, over half of the adolescents (56%) in our study were classified into a profile characterized by high levels of support from all sources, combined with a moderate frequency of online interaction and a low preference for online interaction. These individuals reported the lowest levels of psychosomatic complaints and problematic social media use compared with their counterparts within the other four identified profiles. These findings support an additive model of support, aligning with prior research that has observed a consistent relationship between higher cumulative support from various networks and improved mental health (Chan et al., 2022; Ciarrochi et al., 2017; Jager, 2011; Scholte et al., 2001; Shin & Chang, 2022; Ulmanen, Soini, Pietarinen, & Pyhältö, 2022; Ulmanen, Soini, Pietarinen, Pyhältö, & Rautanen, 2022). Thus, our findings suggest that each source of social support plays a significant role in adolescents' psychosocial well‐being. Nonetheless, disparities within support networks were also evident, as approximately one fifth of our participants (22%) perceived lower support from at least two sources. The highest levels of psychosomatic complaints and problematic social media use were reported by those perceiving low social support from all sources, combined with high online interaction and a strong preference for online interaction over face‐to‐face encounters.

Unlike previous research, we discovered a mixed profile, although small, in which adolescents perceived higher offline support from teachers and classmates but the lowest levels of support from parents and peers across the sample. Earlier studies identified divergent profiles of youth experiencing high levels of support from peers (Chan et al., 2022; Ciarrochi et al., 2017; Shin & Chang, 2022), family or parents (Chan et al., 2022; Ulmanen, Soini, Pietarinen, & Pyhältö, 2022), or a combination of both (Ciarrochi et al., 2017), yet coupled with low support from other sources, or conversely, low support from teachers (Ulmanen, Soini, Pietarinen, & Pyhältö, 2022; Ulmanen, Soini, Pietarinen, Pyhältö, & Rautanen, 2022) alongside high support from alternative sources. Notably, in our study, strong support from educational contexts appeared to provide a partial buffer against the absence of family and peer support. This is reflected by the fact that adolescents in the “Primarily non‐supported online users” profile, who reported moderate support from teachers and classmates, exhibited psychosomatic complaint levels similar to those in the “Primarily supported high online users” profile, who received moderate to high support from all sources. However, it is important to highlight that adolescents in the “Primarily non‐supported online users” profile engaged in less online interaction compared with their peers in the “Primarily supported high online users” profile, who frequently interacted online. This pattern suggests that the degree of online interaction may, to some extent, influence how effectively certain sources of support can compensate for low support from other sources.

Our variable‐centered findings indicated that higher levels of online interaction, particularly with online contacts, and a preference for online interaction over face‐to‐face encounters were associated with poorer psychosocial well‐being. However, our person‐centered analysis added nuance to these results by showing that the relationship between online interactions and psychosocial well‐being is influenced by the level of perceived offline social support. Specifically, individuals who frequently interacted online and experienced high to moderate levels of support across different sources also reported significantly better psychosocial well‐being compared with those who engaged in high levels of online interaction but reported low social support. Although our study did not specifically assess the perceived quality of online interactions, it is possible that online interaction is inherently of lower quality than offline interaction. Previous research on adolescents corroborates this notion, indicating that perceived social support in person serves as a stronger buffer against mental health problems than support provided through online means (Angelini et al., 2022; Lin et al., 2018; Pan et al., 2023). Future research should further investigate the potential disparities in quality between online and offline interactions. Despite the association between online interaction and diminished psychosocial well‐being in our study, it is important not to overlook the potential benefits of online platforms for some individuals, particularly among youth grappling with mental health problems (Pretorius et al., 2019). Prior qualitative research suggests that online interactions can offer positive social engagement that affirms and normalizes emotional experiences, fostering a sense of inclusion and enhancing the ability to navigate distress (Singleton et al., 2016).

Our variable‐centered analysis revealed that interacting online with online contacts, as opposed to offline contacts, was more strongly associated with poorer psychosocial well‐being. Surprisingly, we did not identify a distinct profile characterized by adolescents who highly engaged exclusively in frequent online interactions with one type of interaction partner (offline vs. online contacts). Unlike previous evidence showing that adolescents tend to interact online solely with people known from their offline contexts (Reich et al., 2012), our study makes a significant contribution by revealing that most adolescents who frequently interact online with their offline contacts also engage with online contacts and exhibit a preference for online interaction over face‐to‐face encounters. However, within the profiles, we identified two users—namely “Primarily supported high online users” and “Non‐supported high online users”—were characterized by adolescents who interacted with online contacts either as frequently as, or more often than, with their offline acquaintances. Moreover, those reporting high levels of online interaction generally experienced moderate to high support from various sources, thereby supporting the “rich‐get‐richer” hypothesis, which suggests that individuals with strong social skills and offline social networks benefit more from online interaction, enhancing their existing relationships (Kraut et al., 2002). Our findings also offer limited support for the “social compensation” hypothesis, which suggests that individuals with fewer offline social connections may use online interaction to compensate for their social needs (McKenna et al., 2002). Interestingly, we observed that the majority of participants who perceived high support from all sources tended to engage in moderate levels of online interaction. This contrasts with the previously mentioned hypotheses and indicates a nuanced relationship between online interaction frequency and perceived offline social support.

In our study of Nordic adolescents, we also observed differences in profile membership between countries. Specifically, adolescents from Finland and Iceland were more likely to belong to profiles characterized by lower levels of offline social support and lower levels of psychosocial well‐being compared with the healthiest profile (“Multiply supported online users”). Hence, the importance of social support should be particularly acknowledged in these two countries. It is important to note that there were some differences between the countries in the proportion of participants from different grades in our study. For instance, in Denmark and Norway, a higher proportion of youth were fifth graders compared with the other countries, and they tended to report better psychosocial well‐being than older adolescents, which may have influenced our findings. However, also previous variable‐centered evidence (Lyyra et al., 2021) from the HBSC study shows that youth in Finland and Iceland report poorer mental well‐being and higher loneliness than those living in Denmark and Sweden. In addition, another study (Potrebny et al., 2019) observed that between 2006 and 2014, youth from Finland and Iceland were less likely to report excellent self‐rated health compared with those in the other Nordic countries. Differences in adolescent well‐being between countries could be attributed to various health‐related factors, including large cross‐country variations in socioeconomic inequalities (Gissler et al., 2012; Nielsen et al., 2015) and parental stress (Gunnarsdóttir et al., 2016). Despite the presence of national policies in each Nordic country aimed at reducing social inequalities and child poverty, it has been suggested that they may not adequately support children and families at risk because of ineffective measures and insufficient political commitment to address health inequalities (Eklund Karlsson et al., 2022; Guldbrandsson & Bremberg, 2020).

### Practical implications

Our study has several implications for families, educators, and adolescents. The role of offline social support from multiple sources is crucial in the lives of adolescents. While adolescents derive the most benefit from social support from multiple sources, our study also suggests that improving social support from teachers and classmates, especially for adolescents who perceive low support from other sources, could significantly enhance their psychosocial well‐being. For instance, teachers can be encouraged to interact with students in ways that make them feel valued and to implement strategies in the classroom to foster peer support.

It is important to recognize that adolescents highly engaged in online interaction have the highest risk of problematic social media use, but this risk could be mitigated by high offline social support. Hence, enhancing social support could serve as a strategy to improve the well‐being of these adolescents. Our study also highlights the importance of considering the quality of social networks when assessing the psychosocial health risks associated with adolescents' online engagement.

To reinforce adolescents' ability to navigate online environments, educators could enhance students' social media literacy, potentially mitigating the risks associated with social media use (e.g., exposure to harmful content, interacting with risky contacts, and disclosing excessive personal information) (Potter, 2010; Purington Drake et al., 2023).

### Limitations

This study has several limitations. First, the measures used to assess social support did not specifically differentiate between support perceived through online and offline contexts. Given that these instruments were developed over two decades ago—a period when online interaction was not as widespread among adolescents—these measures may predominantly reflect offline support constructs.

Furthermore, our sample reported high and positively skewed mean ratings of social support. This finding should be carefully considered when interpreting the results, as support levels may vary across different samples. Additionally, profile indicators labeled as “low,” “moderate,” or “high” might be interpreted differently in other contexts. While the mean levels of support in our study may seem elevated, they are not uncommon when compared to existing literature. For instance, the HBSC study, which surveyed adolescents across 42 countries (Bi et al., 2021), found that Nordic countries did not achieve the highest levels in any support measure. This suggests that the support levels observed in our sample, although seemingly high, do not reflect an unusually elevated standard of support when viewed in a broader international context. It is also essential to note that mean values can obscure the underlying variability within the data. Our person‐centered analysis revealed significant differences in support perceptions among adolescents across different profiles. This variability highlights that the overall picture of support in our sample is more complex than mean values alone would suggest.

Another limitation is that the social support measures employed may have captured varying types of support. However, it is important to recognize that different sources of support serve distinct functions. Adolescents often choose their sources of support based on the specific context and nature of their stressors (Skinner & Zimmer‐Gembeck, 2007). For example, they may seek emotional support from family members during personal crises, while turning to classmates or teachers for informational or instrumental assistance in academic situations.

In addition, a concern arises from the cross‐national variability in the scales used to rate family support, which could have influenced the findings. It is also important to note that among the online interaction variables, an ordinal measure—specifically, the frequency of online interaction with online contacts—was treated as a continuous variable to make the online variables comparable and to simplify analysis.

With regard to the LPA used in our study, the interpretive challenges posed by this technique should be acknowledged. Existing literature indicates ambiguity around optimal strategies when determining the most appropriate model (Nylund et al., 2007) and cautions that resultant profiles may not accurately represent distinct subgroups within the population (Williams & Kibowski, 2016). Nevertheless, our approach was based on a considerable sample of Nordic adolescents and aligned with endorsed guidelines (Morin et al., 2016; Woo et al., 2018) to determine the best‐fitting solution.

An additional limitation of our study is its cross‐sectional design, which limits the ability to infer causal relationships between social support, online interactions, and psychosocial well‐being or to track developmental progression within the identified social support profiles. Longitudinal work, such as that of Ciarrochi et al. (2017), has observed that membership in a profile perceived as beneficial in the short term could lead to detrimental long‐term consequences. In light of this, future inquiries would benefit from adopting longitudinal methods to understand the compensatory functions of support and the dynamic transitions of adolescents between social support profiles. Additionally, such research should consider patterns of online interaction to gain a more comprehensive understanding of these processes.

The last notable limitation of our study arises from the timing of our data collection, which took place in 2017 and 2018. Such data may be perceived as outdated given the rapid development of the online landscape. However, it is worth noting that recent evidence (Gustafsson et al., 2023) from Finnish adolescent samples suggests that the intensity of online interaction and the prevalence of problematic social media use have remained consistent from 2018 to 2022. This consistency over the 4‐year period may imply that, contrary to expectations, there has not been a significant shift in these online behaviors.

## CONCLUSION

While the quality of social relationships appears to be more strongly associated with psychosomatic complaints, online interaction behaviors demonstrate a stronger connection to problematic social media use. Adolescents derive the greatest benefit from strong social support across a range of social networks, but support from specific sources can help to counteract the negative effects of low support from other areas. In educational settings, substantial support from teachers and classmates can partially mitigate the lack of family and peer support. Adolescents highly engaged in online interaction report poorer psychosocial well‐being, yet the strength of this link is influenced by their level of offline social support.

## FUNDING INFORMATION

JG was funded by the University of Helsinki and a grant from Nylands Nation.

## CONFLICT OF INTEREST STATEMENT

The authors have no conflicts of interest to declare.

## PATIENT CONSENT STATEMENT

Informed consent was obtained from the participants involved in the study.

## STATEMENT

During the preparation of this work the authors used CurreChat GPT‐4 in order to improve readability and language. After using this tool, the authors reviewed and edited the content as needed and take full responsibility for the content of the publication.

## Supporting information


Data S1.

## Data Availability

The data that support the findings of this study are available from the corresponding author, J. G., upon reasonable request.

## References

[jora13058-bib-0001] Akaike, H. (1987). Factor analysis and AIC. Psychometrika, 52(3), 317–332. 10.1007/BF02294359

[jora13058-bib-0002] Angelini, F. , & Gini, G. (2023). Differences in perceived online communication and disclosing e‐motions among adolescents and young adults: The role of specific social media features and social anxiety. Journal of Adolescence, 96, 512–525. 10.1002/jad.12256 37781933

[jora13058-bib-0003] Angelini, F. , Gini, G. , Marino, C. , & Van Den Eijnden, R. (2024). Social media features, perceived group norms, and adolescents' active social media use matter for perceived friendship quality. Frontiers in Psychology, 15, 1222907. 10.3389/fpsyg.2024.1222907 38721319 PMC11076750

[jora13058-bib-0004] Angelini, F. , Marino, C. , & Gini, G. (2022). Friendship quality in adolescence: The role of social media features, online social support and e‐motions. Current Psychology, 42, 1–17. 10.1007/s12144-022-03564-3 36118141 PMC9465130

[jora13058-bib-0005] Başol, G. (2008). Validity and reliability of the multidimensional scale of perceived social support‐revised, with a Turkish sample. Social Behavior and Personality, 36(10), 1303–1313. 10.2224/sbp.2008.36.10.1303

[jora13058-bib-0006] Best, P. , Manktelow, R. , & Taylor, B. (2014). Online communication, social media and adolescent wellbeing: A systematic narrative review. Children and Youth Services Review, 41, 27–36. 10.1016/j.childyouth.2014.03.001

[jora13058-bib-0007] Beyens, I. , Pouwels, J. L. , van Driel, I. I. , Keijsers, L. , & Valkenburg, P. M. (2020). The effect of social media on well‐being differs from adolescent to adolescent. Scientific Reports, 10(1), 10763. 10.1038/s41598-020-67727-7 32612108 PMC7329840

[jora13058-bib-0008] Bi, S. , Stevens, G. W. J. M. , Maes, M. , Boer, M. , Delaruelle, K. , Eriksson, C. , Brooks, F. M. , Tesler, R. , van der Schuur, W. A. , & Finkenauer, C. (2021). Perceived social support from different sources and adolescent life satisfaction across 42 countries/regions: The moderating role of national‐level generalized trust. Journal of Youth and Adolescence, 50(7), 1384–1409. 10.1007/s10964-021-01441-z 33991276 PMC8219544

[jora13058-bib-0009] Bluemke, M. , Engel, L. , Grüning, D. J. , & Lechner, C. M. (2023). Measuring intellectual curiosity across cultures: Validity and comparability of a new scale in six languages. Journal of Personality Assessment, 106(2), 156–173. 10.1080/00223891.2023.2199863 37125763

[jora13058-bib-0010] Boer, M. , van den Eijnden, R. J. J. M. , Boniel‐Nissim, M. , Wong, S. L. , Inchley, J. C. , Badura, P. , Craig, W. M. , Gobina, I. , Kleszczewska, D. , Klanšček, H. J. , & Stevens, G. W. J. M. (2020). Adolescents' intense and problematic social media use and their well‐being in 29 countries. The Journal of Adolescent Health, 66(6S), S89–S99. 10.1016/j.jadohealth.2020.02.014 32446614 PMC7427320

[jora13058-bib-0011] Boer, M. , van den Eijnden, R. J. J. M. , Finkenauer, C. , Boniel‐Nissim, M. , Marino, C. , Inchley, J. , Cosma, A. , Paakkari, L. , & Stevens, G. W. J. M. (2022). Cross‐national validation of the social media disorder scale: findings from adolescents from 44 countries. Addiction, 117(3), 784–795. 10.1111/add.15709 34605094 PMC7614030

[jora13058-bib-0012] Boniel‐Nissim, M. , van den Eijnden, R. J. , Furstova, J. , Marino, C. , Lahti, H. , Inchley, J. , Šmigelskas, K. , Vieno, A. , & Badura, P. (2022). International perspectives on social media use among adolescents: Implications for mental and social well‐being and substance use. Computers in Human Behavior, 129, 107144. 10.1016/j.chb.2021.107144

[jora13058-bib-0013] Borraccino, A. , Marengo, N. , Dalmasso, P. , Marino, C. , Ciardullo, S. , Nardone, P. , Lemma, P. , & The Hbsc‐Italia Group . (2022). Problematic social media use and cyber aggression in italian adolescents: The remarkable role of social support. International Journal of Environmental Research and Public Health, 19(15), 9763. 10.3390/ijerph19159763 35955121 PMC9367929

[jora13058-bib-0014] Brown, T. A. (2015). Confirmatory factor analysis for applied research (Second Edition). The Guilford Press.

[jora13058-bib-0015] Celeux, G. , & Soromenho, G. (1996). An entropy criterion for assessing the number of clusters in a mixture model. Journal of Classification, 13(2), 195–212. 10.1007/bf01246098

[jora13058-bib-0016] Chan, M. , Sharkey, J. D. , Nylund‐Gibson, K. , Dowdy, E. , & Furlong, M. J. (2022). Social Support Profiles Associations with adolescents' psychological and academic functioning. Journal of School Psychology, 91, 160–177. 10.1016/j.jsp.2022.01.006 35190074

[jora13058-bib-0017] Chen, F. F. (2007). Sensitivity of goodness of fit indexes to lack of measurement invariance. Structural Equation Modeling: A Multidisciplinary Journal, 14(3), 464–504. 10.1080/10705510701301834

[jora13058-bib-0018] Chen, K.‐C. , Liu, S. , Lin, M.‐P. , Lee, Y.‐T. , Wu, J. Y.‐W. , Lin, C.‐A. , & You, J. (2024). A moderated mediation model of the relationship between depression and internet addiction: Mediation by refusal self‐efficacy of internet use and moderation by online and real‐life social support. International Journal of Mental Health and Addiction, 22, 1649–1661. 10.1007/s11469-022-00949-0

[jora13058-bib-0019] Chu, P. S. , Saucier, D. A. , & Hafner, E. (2010). Meta‐analysis of the relationships between social support and well‐being in children and adolescents. Journal of Social and Clinical Psychology, 29(6), 624–645. 10.1521/jscp.2010.29.6.624

[jora13058-bib-0020] Chua, T. H. H. , & Chang, L. (2016). Follow me and like my beautiful selfies: Singapore teenage girls' engagement in self‐presentation and peer comparison on social media. Computers in Human Behavior, 55(Part A), 190–197. 10.1016/j.chb.2015.09.011

[jora13058-bib-0021] Chung, J. E. (2013). Social interaction in online support groups: Preference for online social interaction over offline social interaction. Computers in Human Behavior, 29(4), 1408–1414. 10.1016/j.chb.2013.01.019

[jora13058-bib-0022] Ciarrochi, J. , Morin, A. J. , Sahdra, B. K. , Litalien, D. , & Parker, P. D. (2017). A longitudinal person‐centered perspective on youth social support: Relations with psychological wellbeing. Developmental Psychology, 53(6), 1154–1169. 10.1037/dev0000315 28414510

[jora13058-bib-0023] Clark, S. L. , & Muthén, B. (2009). Relating latent class analysis results to variables not included in the analysis . http://www.statmodel.com/download/relatinglca.pdf

[jora13058-bib-0024] Cohen, J. (1988). Statistical power analysis for the behavioral sciences (2nd ed.). Lawrence Erlbaum Associates.

[jora13058-bib-0025] Cohen, J. (1992). A power primer. Psychological Bulletin, 112(1), 155–159. 10.1037//0033-2909.112.1.155 19565683

[jora13058-bib-0026] Cohen, S. (2004). Social relationships and health. American Psychologist, 59(8), 676–684. 10.1037/0003-066X.59.8.676 15554821

[jora13058-bib-0027] Copeland, W. E. , Wolke, D. , Shanahan, L. , & Costello, E. J. (2015). Adult functional outcomes of common childhood psychiatric problems: A prospective, longitudinal study. JAMA Psychiatry, 72(9), 892–899. 10.1001/jamapsychiatry.2015.0730 26176785 PMC4706225

[jora13058-bib-0028] Cosma, A. , Abdrakhmanova, S. , Taut, D. , Schrijvers, K. , Catunda, C. , & Schnohr, C. (2023). A focus on adolescent mental health and wellbeing in Europe, central Asia and Canada: Health Behaviour in School‐aged Children international report from the 2021/2022 survey: Volume 1. World Health Organization. Regional Office for Europe. https://iris.who.int/handle/10665/373201

[jora13058-bib-0029] Cunningham, S. , Hudson, C. C. , & Harkness, K. (2021). Social media and depression symptoms: A meta‐analysis. Research on Child and Adolescent Psychopathology, 49(2), 241–253. 10.1007/s10802-020-00715-7 33404948

[jora13058-bib-0030] Dahling, J. J. , Gabriel, A. S. , & MacGowan, R. (2017). Understanding typologies of feedback environment perceptions: A latent profile investigation. Journal of Vocational Behavior, 101, 133–148. 10.1016/j.jvb.2017.05.007

[jora13058-bib-0031] de Vries, D. A. , Peter, J. , de Graaf, H. , & Nikken, P. (2016). Adolescents' social network site use, peer appearance‐related feedback, and body dissatisfaction: Testing a mediation model. Journal of Youth and Adolescence, 45(1), 211–224. 10.1007/s10964-015-0266-4 25788122 PMC4698286

[jora13058-bib-0032] Domahidi, E. (2018). The associations between online media use and users' perceived social resources: A meta‐analysis. Journal of Computer‐Mediated Communication, 23(4), 181–200. 10.1093/jcmc/zmy007

[jora13058-bib-0033] Eiroa‐Orosa, F. J. (2020). Understanding psychosocial wellbeing in the context of complex and multidimensional problems. International Journal of Environmental Research and Public Health, 17(16), 5937. 10.3390/ijerph17165937 32824249 PMC7460093

[jora13058-bib-0034] Eklund Karlsson, L. , Balkfors, A. , Gunnarsdottir, H. , Povlsen, L. , Regber, S. , Buch Mejsner, S. , Leena Ikonen, A. , & Fosse, E. (2022). Are universal measures sufficient in reducing child poverty in the Nordic countries? An analysis of policies and political commitments. Scandinavian Journal of Public Health, 50(7), 892–902. 10.1177/14034948221109694 35815562

[jora13058-bib-0035] Fioravanti, G. , Dèttore, D. , & Casale, S. (2012). Adolescent Internet addiction: testing the association between self‐esteem, the perception of Internet attributes, and preference for online social interactions. Cyberpsychology, Behavior and Social Networking, 15(6), 318–323. 10.1089/cyber.2011.0358 22703038

[jora13058-bib-0036] Fischer, R. , & Karl, J. A. (2019). A primer to (Cross‐Cultural) multi‐group invariance testing possibilities in R. Frontiers in Psychology, 10, 1507. 10.3389/fpsyg.2019.01507 31379641 PMC6657455

[jora13058-bib-0037] Floyd, F. J. , & Widaman, K. F. (1995). Factor analysis in the development and refinement of clinical assessment instruments. Psychological Assessment, 7(3), 286–299. 10.1037/1040-3590.7.3.286

[jora13058-bib-0038] Fosse, E. , & Helgesen, M. K. (2019). Policies to address the social determinants of health in the Nordic countries . Report to Nordic Welfare Centre, ISBN: 978‐91‐88213‐47‐1.

[jora13058-bib-0039] Frost, R. L. , & Rickwood, D. J. (2017). A systematic review of the mental health outcomes associated with Facebook use. Computers in Human Behavior, 76, 576–600. 10.1016/j.chb.2017.08.001

[jora13058-bib-0040] Gissler, M. , Rahkonen, O. , Mortensen, L. , Arntzen, A. , Cnattingius, S. , Nybo Andersen, A. M. , & Hemminki, E. (2012). Sex differences in child and adolescent mortality by parental education in the Nordic countries. Journal of Epidemiology and Community Health, 66(1), 57–63. 10.1136/jech.2009.093153 20974838

[jora13058-bib-0041] Grimm, K. J. , Houpt, R. , & Rodgers, D. (2021). Model Fit and Comparison in Finite Mixture Models: A Review and a Novel Approach. Frontiers in Education, 6, 613645. 10.3389/feduc.2021.613645

[jora13058-bib-0042] Guldbrandsson, K. , & Bremberg, S. (2020). Cross‐sectoral cooperation at the ministerial level in three Nordic countries – With a focus on health inequalities. Social Science & Medicine, 256, 112999. 10.1016/j.socscimed.2020.112999 32504865

[jora13058-bib-0043] Gunnarsdóttir, H. , Hensing, G. , Povlsen, L. , & Petzold, M. (2016). Relative deprivation in the Nordic countries‐child mental health problems in relation to parental financial stress. European Journal of Public Health, 26(2), 277–282. 10.1093/eurpub/ckv191 26490511

[jora13058-bib-0044] Gustafsson, J. , Lyyra, N. , Jasinskaja‐Lahti, I. , Simonsen, N. , Lahti, H. , Kulmala, M. , Ojala, K. , & Paakkari, L. (2023). Mental health profiles of Finnish adolescents before and after the peak of the COVID‐19 pandemic. Child and Adolescent Psychiatry and Mental Health, 17(1), 54. 10.1186/s13034-023-00591-1 37120557 PMC10148589

[jora13058-bib-0045] Haugland, S. , & Wold, B. (2001). Subjective health complaints in adolescence‐reliability and validity of survey methods. Journal of Adolescence, 24(5), 611–624. 10.1006/jado.2000.0393 11676508

[jora13058-bib-0046] Heerde, J. A. , & Hemphill, S. A. (2018). Examination of associations between informal help‐seeking behavior, social support, and adolescent psychosocial outcomes: A meta‐analysis. Developmental Review, 47, 44–62. 10.1016/j.dr.2017.10.001

[jora13058-bib-0047] Heinz, A. , Sischka, P. E. , Catunda, C. , Cosma, A. , García‐Moya, I. , Lyyra, N. , Kaman, A. , Ravens‐Sieberer, U. , & Pickett, W. (2022). Item response theory and differential test functioning analysis of the HBSC‐Symptom‐Checklist across 46 countries. BMC Medical Research Methodology, 22(1), 253. 10.1186/s12874-022-01698-3 36175865 PMC9520881

[jora13058-bib-0048] Howard, M. C. , & Hoffman, M. E. (2018). Variable‐centered, person‐centered, and person‐specific approaches: where theory meets the method. Organizational Research Methods, 21(4), 846–876. 10.1177/1094428117744021

[jora13058-bib-0049] Hu, L.‐T. , & Bentler, P. M. (1999). Cutoff criteria for fit indexes in covariance structure analysis: Conventional criteria versus new alternatives. Structural Equation Modeling, 6(1), 1–55. 10.1080/10705519909540118

[jora13058-bib-0050] Inchley, J. , Currie, D. , Cosma, A. , & Samdal, O. (Eds.). (2018). Health behaviour in school‐aged children (HBSC) study protocol: Background, methodology and mandatory items for the 2017/18 survey. CAHRU.

[jora13058-bib-0051] Jager, J. (2011). Convergence and nonconvergence in the quality of adolescent relationships and its association with adolescent adjustment and Young‐Adult Relationship Quality. International Journal of Behavioral Development, 35(6), 497–506. 10.1177/0165025411422992 22334764 PMC3277840

[jora13058-bib-0052] Kamata, A. , Kara, Y. , Patarapichayatham, C. , & Lan, P. (2018). Evaluation of analysis approaches for latent class analysis with auxiliary linear growth model. Frontiers in Psychology, 9, 130. 10.3389/fpsyg.2018.00130 29520242 PMC5826956

[jora13058-bib-0053] Kim, B. , Jee, S. , Lee, J. , An, S. , & Lee, S. M. (2018). Relationships between social support and student burnout: A meta‐analytic approach. Stress and Health, 34(1), 127–134. 10.1002/smi.2771 28639354

[jora13058-bib-0054] Knowles, E. (2024). *Online perceived social support and its association with psychopathology in rural and urban youth* (Doctoral dissertation, University of South Dakota). Dissertations and Theses. https://red.library.usd.edu/diss‐thesis/253

[jora13058-bib-0055] Kraut, R. , Kiesler, S. , Boneva, B. , Cummings, J. N. , Helgeson, V. , & Crawford, A. M. (2002). Internet paradox revisited. Journal of Social Issues, 58(1), 49–74. 10.1111/1540-4560.00248

[jora13058-bib-0056] Larson, J. S. (1996). The World Health Organization's definition of health: Social versus spiritual health. Social Indicators Research, 38(2), 181–192. 10.1007/BF00300458

[jora13058-bib-0057] Laursen, B. P. , & Hoff, E. (2006). Person‐centered and variable‐centered approaches to longitudinal data. Merrill‐Palmer Quarterly, 52(3), 377–389. 10.1353/mpq.2006.0029

[jora13058-bib-0058] Leung, L. (2011). Loneliness, social support, and preference for online social interaction: The mediating effects of identity experimentation online among children and adolescents. Chinese Journal of Communication, 4(4), 381–399. 10.1080/17544750.2011.616285

[jora13058-bib-0059] Lin, M.‐P. , Wu, J. Y.‐W. , You, J. , Chang, K.‐M. , Hu, W.‐H. , & Xu, S. (2018). Association between online and offline social support and internet addiction in a representative sample of senior high school students in Taiwan: The mediating role of self‐esteem. Computers in Human Behavior, 84, 1–7. 10.1016/j.chb.2018.02.007

[jora13058-bib-0060] Lyyra, N. , Junttila, N. , Gustafsson, J. , Lahti, H. , & Paakkari, L. (2022). Adolescents' online communication and well‐being: Findings from the 2018 health behavior in school‐aged children (HBSC) study. Frontiers in Psychiatry, 13, 976404. 10.3389/fpsyt.2022.976404 36276330 PMC9583151

[jora13058-bib-0061] Lyyra, N. , Thorsteinsson, E. B. , Eriksson, C. , Madsen, K. R. , Tolvanen, A. , Löfstedt, P. , & Välimaa, R. (2021). The association between loneliness, mental well‐being, and self‐esteem among adolescents in four Nordic countries. International Journal of Environmental Research and Public Health, 18(14), 7405. 10.3390/ijerph18147405 34299857 PMC8308002

[jora13058-bib-0062] Mascheroni, G. , & Ólafsson, K. (2014). Net Children Go Mobile: Risks and opportunities (Second ed.). Educatt.

[jora13058-bib-0063] McKenna, K. Y. , Green, A. S. , & Gleason, M. E. (2002). Relationship formation on the internet: What's The big attraction? Journal of Social Issues, 58(1), 9–31. 10.1111/1540-4560.00246

[jora13058-bib-0064] McLachlan, G. J. (1987). On bootstrapping the likelihood ratio test statistic for the number of components in a normal mixture. Applied Statistics, 36(3), 318. 10.2307/2347790

[jora13058-bib-0065] Meeus, A. , Beullens, K. , & Eggermont, S. (2022). Social media, unsocial distraction? Testing the associations between preadolescents' SNS use and belonging via two pathways. Media Psychology, 26(4), 363–387. 10.1080/15213269.2022.2147084

[jora13058-bib-0066] Meier, A. , & Reinecke, L. (2021). Computer‐mediated communication, social media, and mental health: A conceptual and empirical meta‐review. Communication Research, 48(8), 1182–1209. 10.1177/0093650220958224

[jora13058-bib-0067] Meyer, J. P. , & Morin, A. J. S. (2016). A person‐centered approach to commitment research: Theory, research, and methodology. Journal of Organizational Behavior, 37(4), 584–612. 10.1002/job.2085

[jora13058-bib-0068] Morgan, G. B. (2015). Mixed mode latent class analysis: An examination of fit index performance for classification. Structural Equation Modeling, 22(1), 76–86. 10.1080/10705511.2014.935751

[jora13058-bib-0069] Morin, A. J. S. , Meyer, J. P. , Creusier, J. , & Biétry, F. (2016). Multiple‐group analysis of similarity in latent profile solutions. Organizational Research Methods, 19(2), 231–254. 10.1177/1094428115621148

[jora13058-bib-0070] Morin, A. J. S. , Morizot, J. , Boudrias, J.‐S. , & Madore, I. (2011). A multifoci person‐centered perspective on workplace affective commitment: A latent profile/factor mixture analysis. Organizational Research Methods, 14(1), 58–90. 10.1177/1094428109356476

[jora13058-bib-0071] Mýlek, V. , Dedkova, L. , & Schouten, A. P. (2024). Adolescents' online communication and self‐disclosure to online and offline acquaintances: Differential effects of social anxiety and depressed moods. Journal of Media Psychology, 36(2), 132–143.

[jora13058-bib-0072] Nakagawa, S. , & Schielzeth, H. (2013). A general and simple method for obtaining R2 from generalized linear mixed‐effects models. Methods in Ecology and Evolution, 4(2), 133–142. 10.1111/j.2041-210x.2012.00261.x

[jora13058-bib-0073] Nesi, J. , Choukas‐Bradley, S. , & Prinstein, M. J. (2018). Transformation of adolescent peer relations in the social media context: Part 1‐A theoretical framework and application to dyadic peer relationships. Clinical Child and Family Psychology Review, 21(3), 267–294. 10.1007/s10567-018-0261-x 29627907 PMC6435354

[jora13058-bib-0074] Nesi, J. , & Prinstein, M. J. (2015). Using social media for social comparison and feedback‐seeking: gender and popularity moderate associations with depressive symptoms. Journal of Abnormal Child Psychology, 43(8), 1427–1438. 10.1007/s10802-015-0020-0 25899879 PMC5985443

[jora13058-bib-0075] Nguyen, H. , & Ho, T. (2022). Online self‐disclosure and well‐being among Vietnamese adolescents: online social support as a mediator. Mental Health and Social Inclusion, 26(4), 339–346. 10.1108/MHSI-01-2022-0003

[jora13058-bib-0076] Nielsen, L. , Damsgaard, M. T. , Meilstrup, C. , Due, P. , Madsen, K. R. , Koushede, V. , & Holstein, B. E. (2015). Socioeconomic differences in emotional symptoms among adolescents in the Nordic countries: recommendations on how to present inequality. Scandinavian Journal of Public Health, 43(1), 83–90. 10.1177/1403494814557885 25377052

[jora13058-bib-0077] Nylund, K. L. , Asparouhov, T. , & Muthén, B. O. (2007). Deciding on the number of classes in latent class analysis and growth mixture modeling: A Monte Carlo simulation study. Structural Equation Modeling, 14(4), 535–569. 10.1080/10705510701575396

[jora13058-bib-0078] Orben, A. (2020). Teenagers, screens and social media: a narrative review of reviews and key studies. Social Psychiatry and Psychiatric Epidemiology, 55(4), 407–414. 10.1007/s00127-019-01825-4 31925481

[jora13058-bib-0079] Orben, A. , Tomova, L. , & Blakemore, S. J. (2020). The effects of social deprivation on adolescent development and mental health. The Lancet. Child & Adolescent Health, 4(8), 634–640. 10.1016/S2352-4642(20)30186-3 32540024 PMC7292584

[jora13058-bib-0080] Pan, Y. , Zhang, Y. , Ma, Z. , Wang, D. , Ross, B. , Huang, S. , & Fan, F. (2023). The more, the better? social capital profiles and adolescent internalizing symptoms: A latent profile analysis. Child Psychiatry and Human Development. Advance online publication. 10.1007/s10578-023-01578-x 37515703

[jora13058-bib-0081] Pastor, D. A. , Barron, K. E. , Miller, B. J. , & Davis, S. L. (2007). A latent profile analysis of college students' achievement goal orientation. Contemporary Educational Psychology, 32(1), 8–47. 10.1016/j.cedpsych.2006.10.003

[jora13058-bib-0082] Peter, J. , & Valkenburg, P. M. (2006). research note: Individual differences in perceptions of internet communication. European Journal of Communication, 21(2), 213–226. 10.1037/0012-1649.43.2.267

[jora13058-bib-0083] Peterson, A. K. , Beymer, P. N. , & Putnam, R. T. (2018). Synchronous and asynchronous discussions: Effects on cooperation, belonging, and affect. Online Learning, 22(4), 7–25. 10.24059/olj.v22i4.1517

[jora13058-bib-0084] Politte‐Corn, M. , Nick, E. A. , & Kujawa, A. (2023). Age‐related differences in social media use, online social support, and depressive symptoms in adolescents and emerging adults. Child and Adolescent Mental Health, 28(4), 497–503. 10.1111/camh.12640 36751140 PMC12051161

[jora13058-bib-0085] Potrebny, T. , Torsheim, T. , Due, P. , Välimaa, R. , Suominen, S. , & Eriksson, C. (2019). Trends in excellent self‐rated health among adolescents: A Comparative Nordic Study. Nordisk Välfärdsforskning | Nordic Welfare Research, 4(2), 67–76. 10.18261/issn.2464-4161-2019-02-04

[jora13058-bib-0086] Potrebny, T. , Wiium, N. , & Lundegård, M. M. (2017). Temporal trends in adolescents' self‐reported psychosomatic health complaints from 1980–2016: A systematic review and meta‐analysis. PLoS One, 12(11), e0188374. 10.1371/journal.pone.0188374 29182644 PMC5705135

[jora13058-bib-0087] Potter, W. J. (2010). The state of media literacy. Journal of Broadcasting & Electronic Media, 54(4), 675–696. 10.1080/08838151.2011.521462

[jora13058-bib-0088] Pretorius, C. , Chambers, D. , & Coyle, D. (2019). Young people's online help‐seeking and mental health difficulties: Systematic narrative review. Journal of Medical Internet Research, 21(11), e13873. 10.2196/13873 31742562 PMC6891826

[jora13058-bib-0089] Prizant‐Passal, S. , Shechner, T. , & Aderka, I. M. (2016). Social anxiety and internet use – a meta‐analysis: What do we know? What are we missing? Computers in Human Behavior, 62, 221–229. 10.1016/j.chb.2016.04.003

[jora13058-bib-0090] Purington Drake, A. , Masur, P. K. , Bazarova, N. N. , Zou, W. , & Whitlock, J. (2023). The Youth Social Media Literacy Inventory: Development and validation using item response theory in the US. Journal of Children and Media, 17(4), 467–487. 10.1080/17482798.2023.2230493

[jora13058-bib-0091] Raphael, D. (2014). Challenges to promoting health in the modern welfare state: The case of the Nordic nations. Scandinavian Journal of Public Health, 42(1), 7–17. 10.1177/1403494813506522 24135426

[jora13058-bib-0092] Ravens‐Sieberer, U. , Erhart, M. , Rajmil, L. , Herdman, M. , Auquier, P. , Bruil, J. , Power, M. , Duer, W. , Abel, T. , Czemy, L. , Mazur, J. , Czimbalmos, A. , Tountas, Y. , Hagquist, C. , Kilroe, J. , & European KIDSCREEN Group . (2010). Reliability, construct and criterion validity of the KIDSCREEN‐10 score: A short measure for children and adolescents' well‐being and health‐related quality of life. Quality of Life Research, 19(10), 1487–1500. 10.1007/s11136-010-9706-5 20668950 PMC2977059

[jora13058-bib-0093] Reich, S. M. , Subrahmanyam, K. , & Espinoza, G. (2012). Friending, IMing, and hanging out face‐to‐face: Overlap in adolescents' online and offline social networks. Developmental Psychology, 48(2), 356–368. 10.1037/a0026980 22369341

[jora13058-bib-0094] Rosenberg, J. M. , Beymer, P. N. , Anderson, D. J. , Van Lissa, C. J. , & Schmidt, J. A. (2018). tidyLPA: An R package to easily carry out latent profile analysis (LPA) using open‐source or commercial software. Journal of Open Source Software, 3(30), 978.

[jora13058-bib-0095] Rosenthal‐von der Pütten, A. M. , Hastall, M. R. , Köcher, S. , Meske, C. , Heinrich, T. , Labrenz, F. , & Ocklenburg, S. (2019). “Likes” as social rewards: Their role in online social comparison and decisions to like other people's selfies. Computers in Human Behavior, 92, 76–86. 10.1016/j.chb.2018.10.017

[jora13058-bib-0096] Rueger, S. Y. , Malecki, C. K. , Pyun, Y. , Aycock, C. , & Coyle, S. (2016). A meta‐analytic review of the association between perceived social support and depression in childhood and adolescence. Psychological Bulletin, 142(10), 1017–1067. 10.1037/bul0000058 27504934

[jora13058-bib-0097] Schlack, R. , Peerenboom, N. , Neuperdt, L. , Junker, S. , & Beyer, A. K. (2021). The effects of mental health problems in childhood and adolescence in young adults: Results of the KiGGS cohort. Journal of Health Monitoring, 6(4), 3–19. 10.25646/8863 PMC8734087 35146318

[jora13058-bib-0098] Scholte, R. H. , Van Lieshout, C. F. , & Van Aken, M. A. (2001). Perceived relational support in adolescence: Dimensions, configurations, and adolescent adjustment. Journal of Research on Adolescence, 11(1), 71–94. 10.1111/1532-7795.00004

[jora13058-bib-0099] Schreurs, L. , Lee, A. Y. , Liu, X. S. , & Hancock, J. T. (2024). When adolescents' self‐worth depends on their social media feedback: A longitudinal investigation with depressive symptoms. Communication Research, 51(6), 631–659. 10.1177/00936502241233787

[jora13058-bib-0100] Schwarz, G. (1978). Estimating the dimension of a model. The Annals of Statistics, 6(2), 461–464. 10.1214/aos/1176344136

[jora13058-bib-0101] Scott, R. A. , Stuart, J. , & Barber, B. L. (2022). Connecting with close friends online: A qualitative analysis of young adults' perceptions of online and offline social interactions with friends. Computers in Human Behavior Reports, 7, 100217. 10.1016/j.chbr.2022.100217

[jora13058-bib-0102] Shin, H. , & Chang, Y. (2022). Relational support from teachers and peers matters: Links with different profiles of relational support and academic engagement. Journal of School Psychology, 92, 209–226. 10.1016/j.jsp.2022.03.006 35618371

[jora13058-bib-0103] Singleton, A. , Abeles, P. , & Smith, I. C. (2016). Online social networking and psychological experiences: The perceptions of young people with mental health difficulties. Computers in Human Behavior, 61, 394–403. 10.1016/j.chb.2016.03.011

[jora13058-bib-0104] Sinha, D. (2011). Concept of psycho‐social well‐being: Western and Indian perspectives. In A. K. Dalal & G. Misra (Eds.), New directions in health psychology (pp. 95–108). Sage Publications.

[jora13058-bib-0105] Skinner, E. A. , & Zimmer‐Gembeck, M. J. (2007). The development of coping. Annual Review of Psychology, 58, 119–144. 10.1146/annurev.psych.58.110405.085705 16903804

[jora13058-bib-0107] Solmi, M. , Radua, J. , Olivola, M. , Croce, E. , Soardo, L. , Salazar de Pablo, G. , Il Shin, J. , Kirkbride, J. B. , Jones, P. , Kim, J. H. , Kim, J. Y. , Carvalho, A. F. , Seeman, M. V. , Correll, C. U. , & Fusar‐Poli, P. (2022). Age at onset of mental disorders worldwide: Large‐scale meta‐analysis of 192 epidemiological studies. Molecular Psychiatry, 27(1), 281–295. 10.1038/s41380-021-01161-7 34079068 PMC8960395

[jora13058-bib-0108] Svetina, D. , Rutkowski, L. , & Rutkowski, D. (2020). Multiple‐group invariance with categorical outcomes using updated guidelines: An illustration using MPLUS and the lavaan/semTOOLS Packages. Structural Equation Modeling: A Multidisciplinary Journal, 27(1), 111–130. 10.1080/10705511.2019.1602776

[jora13058-bib-0109] Tein, J.‐Y. , Coxe, S. , & Cham, H. (2013). Statistical Power to detect the correct number of classes in latent profile analysis. Structural Equation Modeling: A Multidisciplinary Journal, 20(4), 640–657. 10.1080/10705511.2013.824781 24489457 PMC3904803

[jora13058-bib-0110] Thoits, P. A. (1995). Stress, coping, and social support processes: Where are we? What next? Journal of Health and Social Behavior, 35, 53. 10.2307/2626957 7560850

[jora13058-bib-0111] Torsheim, T. , Cavallo, F. , Levin, K. A. , Schnohr, C. , Mazur, J. , Niclasen, B. , & Currie, C. (2015). Psychometric validation of the revised family affluence scale: A latent variable approach. Child Indicators Research, 9(3), 771–784. 10.1007/s12187-015-9339-x 27489572 PMC4958120

[jora13058-bib-0112] Torsheim, T. , Samdal, O. , Rasmussen, M. , Freeman, J. , Griebler, R. , & Dür, W. (2012). Cross‐national measurement invariance of the teacher and classmate support scale. Social Indicators Research, 105(1), 145–160. 10.1007/s11205-010-9770-9 22207777 PMC3229697

[jora13058-bib-0113] Torsheim, T. , Wold, B. , & Samdal, O. (2000). The teacher and classmate support scale. School Psychology International, 21(2), 195–212. 10.1177/0143034300212006

[jora13058-bib-0115] Ulmanen, S. , Soini, T. , Pietarinen, J. , & Pyhältö, K. (2022). Development of students' social support profiles and their association with students' study wellbeing. Developmental Psychology, 58(12), 2336–2349. 10.1037/dev0001439 36048099

[jora13058-bib-0116] Ulmanen, S. , Soini, T. , Pietarinen, J. , Pyhältö, K. , & Rautanen, P. (2022). Primary and lower secondary school students' social support profiles and study wellbeing. The Journal of Early Adolescence, 42(5), 613–646. 10.1177/02724316211058061

[jora13058-bib-0117] UNICEF . (2021). The state of the world's children: On my mind. Promoting, protecting and caring. Regional brief: Europe . https://www.unicef.org/eu/reports/state‐worlds‐children‐2021. Accessed 23 Apr 2024

[jora13058-bib-0118] Valkenburg, P. M. , Meier, A. , & Beyens, I. (2022). Social media use and its impact on adolescent mental health: An umbrella review of the evidence. Current Opinion in Psychology, 44, 58–68. 10.1016/j.copsyc.2021.08.017 34563980

[jora13058-bib-0119] van de Schoot, R. , Lugtig, P. , & Hox, J. (2012). A checklist for testing measurement invariance. European Journal of Developmental Psychology, 9(4), 486–492.

[jora13058-bib-0120] Van den Eijnden, R. J. J. M. , Lemmens, J. S. , & Valkenburg, P. M. (2016). The Social Media Disorder Scale. Computers in Human Behavior, 61, 478–487. 10.1016/j.chb.2016.03.038

[jora13058-bib-0121] van Duin, C. , Heinz, A. , & Willems, H. (2021). Predictors of problematic social media use in a nationally representative sample of adolescents in Luxembourg. International Journal of Environmental Research and Public Health, 18(22), 11878. 10.3390/ijerph182211878 34831633 PMC8619406

[jora13058-bib-0122] Walther, J. B. (1996). Computer‐mediated communication: Impersonal, interpersonal, and hyperpersonal interaction. Communication Research, 23(1), 3–43. 10.1177/009365096023001001

[jora13058-bib-0123] Williams, G. A. , & Kibowski, F. (2016). Latent class analysis and latent profile analysis. In L. A. Jason & D. S. Glenwick (Eds.), Handbook of methodological approaches to community‐based research: Qualitative, quantitative, and mixed methods (pp. 143–151). Oxford University Press.

[jora13058-bib-0124] Wohn, D. Y. , Carr, C. T. , & Hayes, R. A. (2016). How affective is a “like”?: The effect of paralinguistic digital affordances on perceived social support. Cyberpsychology, Behavior, and Social Networking, 19(9), 562–566. 10.1089/cyber.2016.0162 27635443

[jora13058-bib-0125] Wong, S. L. , King, N. , Gariépy, G. , Michaelson, V. , Canie, O. , King, M. , Craig, W. , & Pickett, W. (2022). Adolescent social media use and its association with relationships and connections: Canadian Health Behaviour in School‐aged Children, 2017/2018. Health Reports, 33(12), 14–23. 10.25318/82-003-x202201200002-eng 36542360

[jora13058-bib-0126] Woo, S. E. , Jebb, A. T. , Tay, L. , & Parrigon, S. (2018). Putting the “Person” in the center: Review and synthesis of person‐centered approaches and methods in organizational science. Organizational Research Methods, 21(4), 814–845.

[jora13058-bib-0127] Wu, H. , & Estabrook, R. (2016). Identification of confirmatory factor analysis models of different levels of invariance for ordered categorical outcomes. Psychometrika, 81(4), 1014–1045. 10.1007/s11336-016-9506-0 27402166 PMC5458787

[jora13058-bib-0128] Yau, J. C. , & Reich, S. M. (2018). Are the qualities of adolescents' Offline friendships present in digital interactions? Adolescent Research Review, 3, 339–355. 10.1007/s40894-017-0059-y

[jora13058-bib-0129] Zhou, Z. , & Cheng, Q. (2022). Relationship between online social support and adolescents' mental health: A systematic review and meta‐analysis. Journal of Adolescence, 94(3), 281–292. 10.1002/jad.12031 35390193

[jora13058-bib-0130] Zimet, G. D. , Dahlem, N. W. , Zimet, S. G. , & Farley, G. K. (1988). Multidimensional scale of perceived social support. Journal of Personality Assessment, 52(1), 30–41. 10.1207/s15327752jpa5201_2 2280326

