# Supplementary material for: Social support and online interaction and their links to psychosocial well‐being among Nordic adolescents: Integrating variable‐centered and person‐centered approaches
Source: J Res Adolesc. 2025 Jan 24;35(1):e13058. doi: 10.1111/jora.13058 (PMC11758761; doi:10.1111/jora.13058)
Supplement: Supplementary file 1 — Data S1. [file JORA-35-0-s001.docx]

**Supplementary Table 1.** Fit indices for measurement invariance of the preference for and intensity of online interaction.

|  | scaled *χ^2^* | df | scaled CFI | scaled TLC | scaled RMSEA | scaled SRMR |
| --- | --- | --- | --- | --- | --- | --- |
| Configural invariance model | 304.94 | 40 | 0.999 | 0.999 | 0.041 | 0.020 |
| Threshold invariance model | 724.69 | 100 | 0.999 | 0.999 | 0.040 | 0.020 |
| Metric invariance model | 869.31 | 116 | 0.998 | 0.999 | 0.042 | 0.024 |
| Scalar invariance model | 1884.90 | 132 | 0.996 | 0.998 | 0.059 | 0.025 |

*Note.* χ^2^, chi-square; df, degrees of freedom; CFI, comparative fit index; TLI, Tucker-Lewis index; RMSEA, root mean square error of approximation; SRMR, standardized root mean square residual.

**Supplementary Table 2.** Results from the mixed-effect linear regression showing associations between sociodemographic characteristics and psychosocial well-being.

|  |  | Psychosomatic complaints  (*n* =17568–20070) | | | | Problematic social media use  (*n* = 17401–18633) | | | |
| --- | --- | --- | --- | --- | --- | --- | --- | --- | --- |
|  |  | B (95% CI) | β | *p*-value | Marginal pseudo *R*^2^ | B (95% CI) | β | *p*-value | Marginal pseudo *R*^2^ |
| Gender, female (reference male) | Adjusted **a** | **0.36 (0.33, 0.38)** | **0.42** | **<.001** | .104 | **0.37 (0.31, 0.43)** | **0.18** | **<.001** | .043 |
|  | Adjusted **b** | **0.35 (0.33, 0.38)** | **0.41** | **<.001** | .237 | **0.42 (0.36, 0.47)** | **0.20** | **<.001** | .139 |
| Grade (reference 5) |  |  |  |  |  |  |  |  |  |
| 7th | Adjusted **a** | **0.12 (0.09, 0.15)** | **0.14** | **<.001** |  | **0.35 (0.27, 0.43)** | **0.17** | **<.001** |  |
|  | Adjusted **b** | −0.02 (−0.05, 0.01) | −0.03 | .108 |  | −0.04 (−0.12, 0.04) | −0.02 | .341 |  |
| 9th | Adjusted **a** | **0.26 (0.23, 0.29)** | **0.30** | **<.001** |  | **0.45 (0.37, 0.53)** | **0.21** | **<.001** |  |
|  | Adjusted **b** | **0.06 (0.03, 0.09)** | **0.07** | **<.001** |  | **−0.11 (−0.19, −0.02)** | **−0.05** | **.012** |  |
| Family affluence | Adjusted **a** | **−0.01 (−0.02, −0.01)** | **−0.03** | **<.001** |  | 0.01 (−0.01, 0.03) | 0.01 | .228 |  |
|  | Adjusted **b** | **−0.01 (−0.01, 0.00)** | **−0.01** | **.042** |  | 0.00 (−0.01, 0.02) | 0.00 | .746 |  |
| Family structure (reference nuclear family) | |  |  |  |  |  |  |  |  |
| Single-parent family | Adjusted **a** | **0.21 (0.18, 0.25)** | **0.25** | **<.001** |  | **0.31 (0.23, 0.39)** | **0.15** | **<.001** |  |
|  | Adjusted **b** | **0.13 (0.10, 0.16)** | **0.15** | **<.001** |  | **0.15 (0.07, 0.23)** | **0.07** | **<.001** |  |
| Stepfamily | Adjusted **a** | **0.18 (0.15, 0.22)** | **0.22** | **<.001** |  | **0.26 (0.17, 0.36)** | **0.13** | **<.001** |  |
|  | Adjusted **b** | **0.11 (0.08, 0.15)** | **0.13** | **<.001** |  | **0.12 (0.03, 0.22)** | **0.06** | **.007** |  |
| Foster care or children’s home | Adjusted **a** | **0.31 (0.20, 0.42)** | **0.36** | **<.001** |  | **0.82 (0.52, 1.13)** | **0.39** | **<.001** |  |
|  | Adjusted **b** | **0.12 (0.01, 0.24)** | **0.14** | **.037** |  | **0.58 (0.27, 0.89)** | **0.28** | **<.001** |  |
| Immigrant background (reference native) | |  |  |  |  |  |  |  |  |
| First-generation | Adjusted **a** | **−0.07 (−0.11, −0.03)** | **−0.09** | **<.001** |  | **0.38 (0.27, 0.49)** | **0.18** | **<.001** |  |
|  | Adjusted **b** | **−0.10 (−0.14, −0.06)** | **−0.11** | **<.001** |  | **0.35 (0.24, 0.46)** | **0.17** | **<.001** |  |
| Second-generation | Adjusted **a** | 0.02 (−0.01, 0.05) | 0.02 | .230 |  | **0.18 (0.09, 0.26)** | **0.08** | **<.001** |  |
|  | Adjusted **b** | −0.01 (−0.05, 0.02) | −0.02 | .386 |  | **0.14 (0.06, 0.23)** | **0.07** | **.001** |  |
| Country (reference Finland) |  |  |  |  |  |  |  |  |  |
| Denmark | Adjusted **a** | **−0.22 (−0.28, −0.16)** | **−0.26** | **<.001** |  | **−0.56 (−0.80, −0.31)** | **−0.27** | **<.001** |  |
|  | Adjusted **b** | **−0.14 (−0.19, −0.08)** | **−0.16** | **<.001** |  | **−0.44 (−0.67, −0.20)** | **−0.21** | **<.001** |  |
| Iceland | Adjusted **a** | −0.02 (−0.07, 0.03) | −0.03 | .371 |  | **−0.74 (−0.94, −0.53)** | **−0.35** | **<.001** |  |
|  | Adjusted **b** | 0.04 (0.00, 0.09) | 0.05 | .054 |  | **−0.65 (−0.85, −0.46)** | **−0.31** | **<.001** |  |
| Norway | Adjusted **a** | **−0.25 (−0.31, −0.20)** | **−0.30** | **<.001** |  | 0.01 (−0.18, 0.20) | 0.00 | .953 |  |
|  | Adjusted **b** | **−0.13 (−0.17, −0.08)** | **−0.15** | **<.001** |  | 0.16 (−0.02, 0.34) | 0.08 | .080 |  |
| Sweden | Adjusted **a** | **0.08 (0.04, 0.13)** | **0.10** | **<.001** |  | **−0.44 (−0.62, −0.26)** | **−0.21** | **<.001** |  |
|  | Adjusted **b** | **0.17 (0.13, 0.21)** | **0.20** | **<.001** |  | **−0.36 (−0.53, −0.18)** | **−0.17** | **<.001** |  |

*Note*. B, unstandardized regression coefficient; CI, confidence interval; β, standardized regression coefficient. Marginal *R*^2^ represents the variance explained by independent variables using *R*^2^ statistics of Nakagawa and Schielzeth (2013). Bold values denote statistical significance.

**a** Adjusted for sociodemographic characteristics (gender, grade, family affluence, family structure, immigrant background, and country).

**b** Adjusted for sociodemographic characteristics, social support, and online variables (preference for online interaction, and intensity of online interaction).

|  | All (100%) | Profile 1 –“Multiply supported online users” (56%) | Profile 2 –”Primarily supported high online users” (22%) | Profile 3 –“Non-supported online users” (13%) | Profile 4 –“Primarily non-supported online users” (5%) | Profile 5 – “Non-supported high online users” (4%) | Profile comparison | | |
| --- | --- | --- | --- | --- | --- | --- | --- | --- | --- |
|  | *n* = 18057–18659 | *n* = 10149–10476 | *n* = 3966–4100 | *n* = 2274–2351 | *n* = 901–935 | *n* = 767–797 | Overall | | Pairwise comparison |
|  | % / *M* (*SD*) | % / *M* (*SD*) | % / *M* (*SD*) | % / *M* (*SD*) | % / *M* (*SD*) | % / *M* (*SD*) | χ2 / *F* | *p*-value |  |
| Gender, female (vs male) | 52.0 | 53.7 | 46.4 | 53.0 | 51.5 | 56.3 | 66.23 | <.001 | 1 and 2**^a^**, 2 and 3 **^a^**, 2 and 5**^a^** differ |
| Grade |  |  |  |  |  |  | 1175.96 | <.001 |  |
| 5th | 33.7 | 43.2 | 19.1 | 26.1 | 26.3 | 10.6 |  |  | 1 and 2**^a^**, 1 and 3**^a^**, 1 and 4**^a^**, 1 and 5**^a^**, 2 and 3**^a^**, 2 and 4**^a^**, 2 and 5**^a^**, 3 and 5**^a^**, 4 and 5**^a^** differ |
| 7th | 34.2 | 32.0 | 36.9 | 35.8 | 41.0 | 37.4 |  |  | 1 and 2**^a^**, 1 and 3**^c^**, 1 and 4**^a^**, 1 and 5**^c^** differ |
| 9th | 32.1 | 24.8 | 44.0 | 38.1 | 32.7 | 52.0 |  |  | 1 and 2**^a^**, 1 and 3**^a^**, 1 and 4**^a^**, 1 and 5**^a^**, 2 and 3**^a^**, 2 and 4**^a^**, 2 and 5**^b^**, 3 and 4**^c^**, 3 and 5**^a^**, 4 and 5**^a^** differ |
| Family affluence | 9.35 (1.86) | 9.42 (1.81) | 9.50 (1.87) | 8.93 (1.93) | 9.04 (2.00) | 8.99 (1.92) | 49.67 | <.001 | 1 and 3**^a^**, 1 and 4**^a^**, 1 and 5**^a^**, 2 and 3**^a^**, 2 and 4**^a^**, 2 and 5**^a^** differ |
| Family structure |  |  |  |  |  |  | 291.07 | <.001 |  |
| Nuclear family | 72.5 | 76.9 | 70.0 | 63.7 | 66.1 | 57.7 |  |  | 1 and 2**^a^**, 1 and 3**^a^**, 1 and 4**^a^**, 1 and 5**^a^**, 2 and 3**^a^**, 2 and 5**^a^**, 3 and 5**^c^**, 4 and 5**^b^** differ |
| Single-parent family | 15.5 | 13.2 | 16.5 | 20.5 | 17.9 | 25.0 |  |  | 1 and 2**^a^**, 1 and 3**^a^**, 1 and 4**^b^**, 1 and 5**^a^**, 2 and 3**^b^**, 2 and 5**^a^**, 4 and 5**^b^** differ |
| Stepfamily | 11.0 | 9.3 | 12.6 | 14.2 | 14.2 | 15.2 |  |  | 1 and 2**^a^**, 1 and 3**^a^**, 1 and 4**^a^**, 1 and 5**^a^** differ |
| Foster care or  children’s home | 0.9 | 0.6 | 1.0 | 1.6 | 0.9 | 2.1 |  |  | 1 and 3**^a^**, 1 and 5**^a^** differ |
| Immigrant background |  |  |  |  |  |  | 20.473 | <.001 |  |
| First-generation  immigrant | 7.8 | 7.3 | 8.1 | 8.7 | 9.1 | 9.7 |  |  | No differences |
| Second-generation  immigrant | 14.2 | 13.8 | 14.3 | 15.6 | 13.6 | 15.5 |  |  | No differences |
| Native (non-immigrant) | 78.0 | 79.0 | 77.6 | 75.7 | 77.3 | 74.8 |  |  | 1 and 3**^c^** differ |
| Country |  |  |  |  |  |  | 873.08 | <.001 |  |
| Denmark | 16.7 | 17.6 | 18.4 | 19.1 | 1.7 | 7.4 |  |  | 1 and 4**^a^**, 1 and 5**^a^**, 2 and 4**^a^**, 2 and 5**^a^**, 3 and 4**^a^**, 3 and 5**^a^**, 4 and 5**^a^** differ |
| Finland | 16.3 | 14.1 | 18.3 | 20.5 | 17.9 | 22.7 |  |  | 1 and 2**^a^**, 1 and 3**^a^**, 1 and 4**^c^**, 1 and 5**^a^**, 2 and 5**^c^** differ |
| Iceland | 32.6 | 31.6 | 26.8 | 29.9 | 67.1 | 42.5 |  |  | 1 and 2**^a^**, 1 and 4**^a^**, 1 and 5**^a^**, 2 and 4**^a^**, 2 and 5**^a^**, 3 and 4**^a^**, 3 and 5**^a^**, 4 and 5**^a^** differ |
| Norway | 15.5 | 18.0 | 14.6 | 12.4 | 3.8 | 9.4 |  |  | 1 and 2**^a^**, 1 and 3**^a^**, 1 and 4**^a^**, 1 and 5**^a^**, 2 and 4**^a^**, 2 and 5**^b^**, 3 and 4**^a^**, 4 and 5**^a^** differ |
| Sweden | 18.8 | 18.7 | 22.0 | 18.0 | 9.6 | 18.1 |  |  | 1 and 2**^a^**, 1 and 4**^a^**, 2 and 3**^b^**, 2 and 4**^a^**, 3 and 4**^a^**, 4 and 5**^a^** differ |
| Family support | 5.94 (1.54) | 6.66 (0.48) | 6.41 (0.69) | 4.56 (0.79) | 1.49 (0.62) | 2.83 (1.19) | 22670.15 | <.001 | All differ **^a^** |
| Peer support | 5.62 (1.62) | 6.10 (1.12) | 6.06 (1.18) | 4.54 (1.53) | 2.20 (1.48) | 3.92 (2.09) | 2862.86 | <.001 | 1 and 3**^a^**, 1 and 4**^a^**, 1 and 5**^a^**, 2 and 3**^a^**, 2 and 4**^a^**, 2 and 5**^a^**, 3 and 4**^a^**, 3 and 5**^a^**, 4 and 5**^a^** differ |
| Teacher support | 4.04 (0.87) | 4.27 (0.73) | 3.97 (0.90) | 3.38 (0.85) | 3.98 (0.89) | 3.29 (1.04) | 707.41 | <.001 | 1 and 2**^a^**, 1 and 3**^a^**, 1 and 4**^a^**, 1 and 5**^a^**, 2 and 3**^a^**, 2 and 5**^a^**, 3 and 4**^a^**, 4 and 5**^a^** differ |
| Classmate support | 4.01 (0.76) | 4.15 (0.69) | 4.03 (0.76) | 3.45 (0.76) | 3.94 (0.79) | 3.59 (0.88) | 459.59 | <.001 | 1 and 2**^a^**, 1 and 3**^a^**, 1 and 4**^a^**, 1 and 5**^a^**, 2 and 3**^a^**, 2 and 4**^b^**, 2 and 5**^a^**, 3 and 4**^a^**, 3 and 5**^b^**, 4 and 5**^a^** differ |
| Preference for online interaction | 2.49 (1.21) | 2.20 (1.12) | 2.96 (1.23) | 2.76 (1.15) | 2.43 (1.19) | 3.27 (1.25) | 428.37 | <.001 | All differ **^a^** |
| Intensity of online interaction with  “offline contacts” | 3.06 (1.04) | 2.87 (0.98) | 3.78 (0.88) | 2.57 (0.90) | 2.79 (0.99) | 3.70 (0.97) | 888.58 | <.001 | 1 and 2**^a^**, 1 and 3**^a^**, 1 and 5**^a^**,2 and 3**^a^**, 2 and 4**^a^**, 3 and 4**^a^**, 3 and 5**^a^**, 4 and 5**^a^** differ |
| Intensity of online interaction with  “online contacts” | 1.94 (1.34) | 1.16 (0.37) | 3.93 (0.82) | 1.46 (0.65) | 1.38 (0.66) | 4.27 (0.74) | 20473.05 | <.001 | 1 and 2**^a^**, 1 and 3**^a^**, 1 and 4**^a^**, 1 and 5**^a^**, 2 and 3**^a^**, 2 and 4**^a^**, 2 and 5**^a^**, 3 and 4^b^, 3 and 5**^b^**, 4 and 5**^a^** differ |

**Supplementary Table 3.** Distribution of sociodemographic characteristics and indicator variables across the identified profiles.

*Note.* Chi-square test for percentage comparison and post-hoc analysis of variance for mean comparison (two-tailed). Scores ranged from 0 to 13 for family affluence, 1 to 7 for family and peer support, 1 to 5 for teacher and classmate support, 1 to 3 for preference for online interaction, and 1 to 5 for intensity of online interaction. The data are weighted by the posterior probabilities of the units for the 5-class solution.

^a^ Profiles were significantly different from each other (*p* < .001) using Bonferroni-corrected pairwise comparisons.

^b^ Profiles were significantly different from each other (*p* < .01) using Bonferroni-corrected pairwise comparisons.

^c^ Profiles were significantly different from each other (*p* < .05) using Bonferroni-corrected pairwise comparisons.

**Supplementary Figure 1.** Line graphs indicating different profile solutions (Models 1 and 3) showing standardized mean values on the y-axis and the profile indicators on the x-axis.


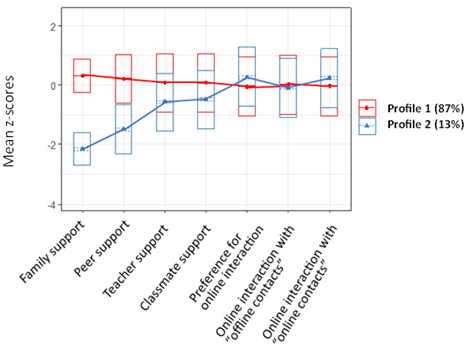

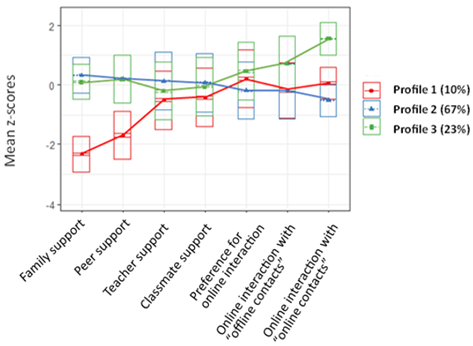
Model 1, 2-class solution Model 1, 3-class solution


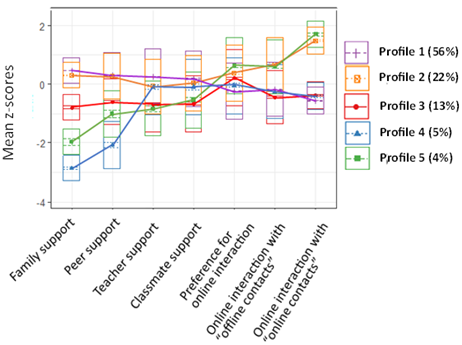

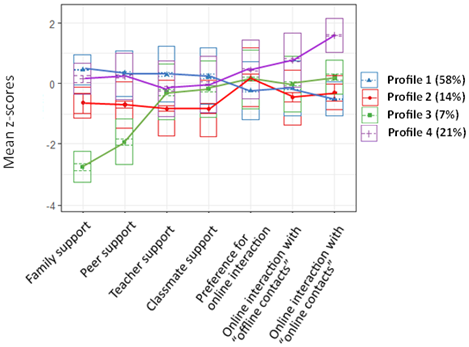
Model 1, 4-class solution Model 1, 5-class solution


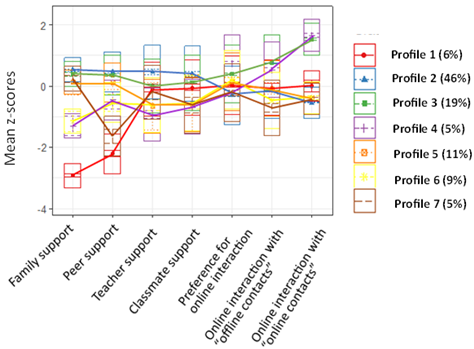

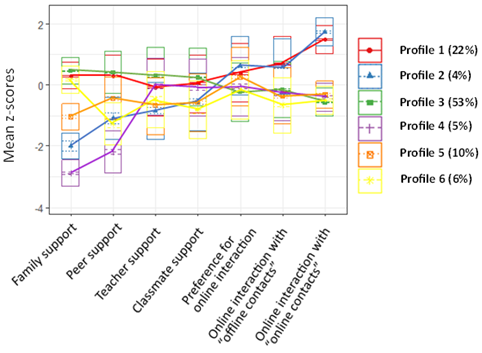
Model 1, 6-class solution Model 1, 7-class solution


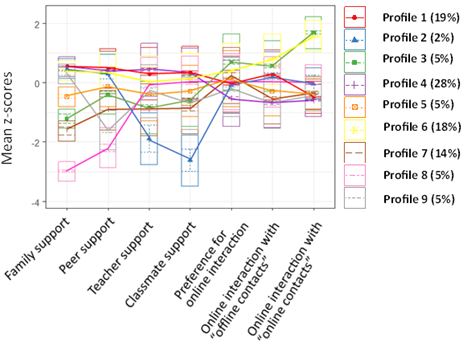

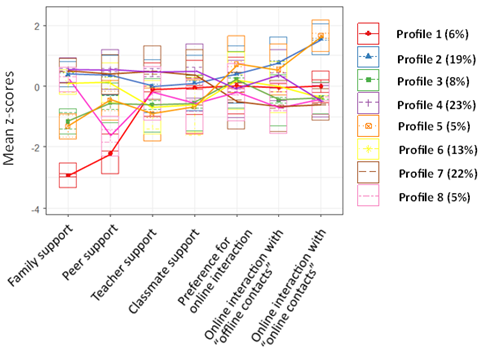
Model 1, 8-class solution Model 1, 9-class solution


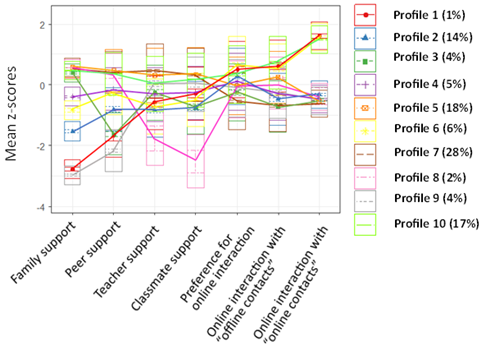
Model 1, 10-class solution


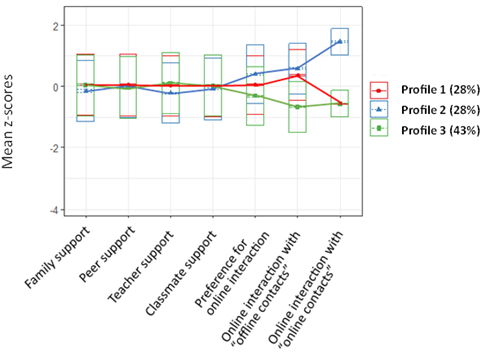

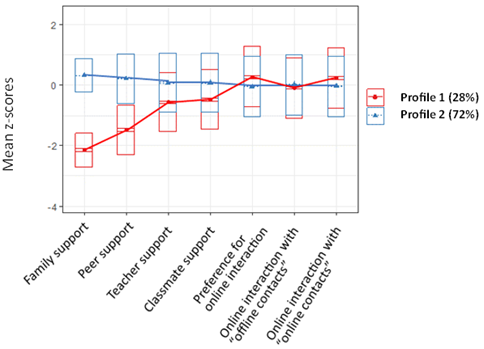
Model 3, 2-class solution Model 3, 3-class solution


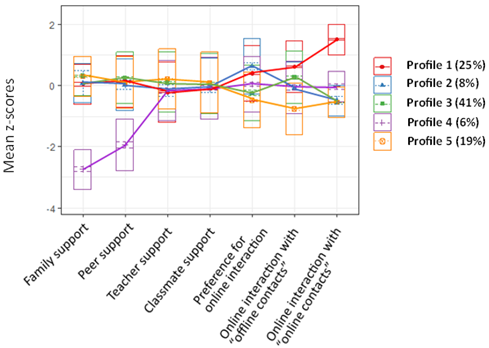

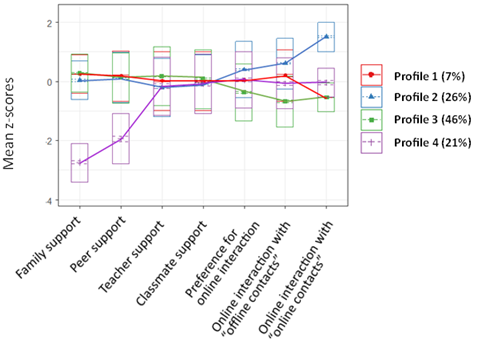
Model 3, 4-class solution Model 3, 5-class solution


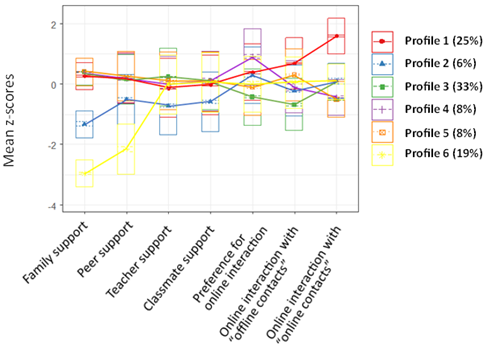
Model 3, 6-class solution

The 7- to 10-class solutions for Model 3 were not plotted as the entropy value was considered low and the models resulted in warning messages (“One or more analyses resulted in warnings! Examine these analyses carefully: model_3_class_7, model_3_class_8, model_3_class_9, model_3_class_10”).

**Supplementary Figure 2.** Bar graphs showing the prevalence of problematic social media users across the identified profiles when adjusting for covariates and the nested structure of the data.

*Note*. A score of 6 or more on a scale from 0 to 9 was set as the threshold for classifying individuals as problematic social media users. Bars represent 95% confidence intervals for the proportion of problematic social media users. The data are weighted by the posterior probabilities of the units for the 5-class solution.
